# Penetrating Exploration of Prognostic Correlations of the FKBP Gene Family with Lung Adenocarcinoma

**DOI:** 10.3390/jpm13010049

**Published:** 2022-12-26

**Authors:** Chin-Chou Wang, Wan-Jou Shen, Gangga Anuraga, Yu-Hsiu Hsieh, Hoang Dang Khoa Ta, Do Thi Minh Xuan, Chiu-Fan Shen, Chih-Yang Wang, Wei-Jan Wang

**Affiliations:** 1 Divisions of Pulmonary & Critical Care Medicine, Department of Internal Medicine, Kaohsiung Chang Gung Memorial Hospital, Chang Gung University College of Medicine, Kaohsiung 83301, Taiwan; 2 Department of Respiratory Therapy, Kaohsiung Chang Gung Memorial Hospital, Chang Gung University College of Medicine, Kaohsiung 83301, Taiwan; 3 Department of Respiratory Care, Chang Gung University of Science and Technology, Chiayi 613016, Taiwan; 4 Department of Biological Science and Technology, China Medical University, Taichung 40676, Taiwan; 5 Graduate Institute of Cancer Biology and Drug Discovery, College of Medical Science and Technology, Taipei Medical University, Taipei 11031, Taiwan; 6 Ph.D. Program for Cancer Molecular Biology and Drug Discovery, College of Medical Science and Technology, Taipei Medical University and Academia Sinica, Taipei 11031, Taiwan; 7 Department of Statistics, Faculty of Science and Technology, Universitas PGRI Adi Buana, Surabaya 60234, East Java, Indonesia; 8 TMU Research Center of Cancer Translational Medicine, Taipei Medical University, Taipei 11031, Taiwan; 9 Research Center for Cancer Biology, China Medical University, Taichung 40676, Taiwan

**Keywords:** *FKBP* family genes, lung cancer, metabolism, prognosis, bioinformatics

## Abstract

The complexity of lung adenocarcinoma (LUAD), the development of which involves many interacting biological processes, makes it difficult to find therapeutic biomarkers for treatment. FK506-binding proteins (FKBPs) are composed of 12 members classified as conservative intracellular immunophilin family proteins, which are often connected to cyclophilin structures by tetratricopeptide repeat domains and have peptidyl prolyl isomerase activity that catalyzes proline from residues and turns the trans form into the cis form. Since FKBPs belong to chaperone molecules and promote protein folding, previous studies demonstrated that FKBP family members significantly contribute to the degradation of damaged, misfolded, abnormal, and foreign proteins. However, transcript expressions of this gene family in LUAD still need to be more fully investigated. In this research, we adopted high-throughput bioinformatics technology to analyze *FKBP* family genes in LUAD to provide credible information to clinicians and promote the development of novel cancer target drugs in the future. The current data revealed that the messenger (m)RNA levels of *FKBP2*, *FKBP3*, *FKBP4*, *FKBP10*, *FKBP11*, and *FKBP14* were overexpressed in LUAD, and *FKBP10* had connections to poor prognoses among LUAD patients in an overall survival (OS) analysis. Based on the above results, we selected *FKBP10* to further conduct a comprehensive analysis of the downstream pathway and network. Through a DAVID analysis, we found that *FKBP10* was involved in mitochondrial electron transport, NADH to ubiquinone transport, mitochondrial respiratory chain complex I assembly, etc. The MetaCore pathway analysis also indicated that *FKBP10* was involved in "Ubiquinone metabolism", "Translation_(L)-selenoaminoacid incorporation in proteins during translation", and "Transcription_Negative regulation of HIF1A function". Collectively, this study revealed that *FKBP* family members are both significant prognostic biomarkers for lung cancer progression and promising clinical therapeutic targets, thus providing new targets for treating LUAD patients.

## 1. Introduction

In its advanced stages, lung cancer (LC) is the one of the most lethal types of cancer and is responsible for significant mortality worldwide. The 5-year overall survival (OS) of patients with LC ranges from 5% to 17.7% [1]. Depending on the histology, LC includes small cell lung carcinoma (SCLC) and non-SCLC (NSCLC) subtypes. NSCLC is further divided into three secondary categories: lung adenocarcinoma (LUAD), squamous cell lung carcinoma (SCLC), and large cell lung carcinoma (LCLC). However, the proportion of LUAD is up to 40%, thereby representing the main form of LC. The leading cause of LUAD, which originates from the small airway epithelium and the secretion of mucus and other substances from type II alveolar cells, is smoking. Additionally, LUAD easily occurs in the periphery of the lungs. Due to its early spread, aggression, and ability to easily metastasize, the survival rate of LUAD is less than 5 years [2,3,4]. Although several therapies such as chemotherapy and surgical resection are used to decrease mortality, there are still dilemmas including late detection for approximately 75% of LUAD patients, as well as high recurrence rates and poor prognoses [5,6]. Therefore, it is imperative to explore potential biomarkers and develop novel targeted cancer therapies for future application [7,8,9,10,11]. 

FK506-binding proteins (FKBPs) include 12 members and are classified as conservative intracellular immunophilin family proteins. FKBPs are often connected to cyclophilin structures via tetratricopeptide repeat domains and have peptidyl prolyl isomerase activity, which catalyzes proline from residues to turn the trans form into the cis form [12,13,14,15]. FKBPs are chaperone molecules and promote protein folding [16]. FKBP family members, moreover, are tied to immunosuppressants such as FK506, rapamycin, and cyclosporin A to induce multiple physiological responses [12]. Thus, these proteins are related to numerous human malignancies. As an example, FKBP2 is overexpressed in the hypoxic environment of glioblastoma multiforme and triggers tumor metastasis [17]. FKBP3 was reported to be closely associated with colorectal cancer [18] and NSCLC [19]. FKBP4 expression was also found to be upregulated in breast cancer [20], in NSCLC by activating the Akt-mammalian target of rapamycin (mTOR) signaling pathway [21], and in colorectal cancer in males [22]. Notably, FKBP5 may become a prognostic indicator in pancreatic cancer [23]. The methylation of FKBP6 was found to serve as a novel biomarker in cervical cancer [24]. Previous studies reported that transcription levels of FKBP7 were increased in prostate cancer [25] and melanomas [26]. FKBP9 was proven to be related to poor prognoses in prostate cancer [27] and gliomas [28]. Transcriptional levels of FKBP10 and FKBP11 are upregulated in renal cell carcinoma tissues compared to normal tissues [13], and FKBP14 is involved in various types of human tumors such as gastric cancer [29,30], human cervical cancers [31], ovarian cancer [32], and osteosarcomas [33]. In addition, FKBP15 was verified to be upregulated in breast cancer patients [34]. Although the *FKBP* gene family has been studied in a variety of human malignancies, a comprehensive analysis including individual gene expression levels, genetic variations, immune infiltration in the tumor microenvironment (TME), and biological mechanisms of *FKBP* family members in LUAD has not been elaborated [35,36,37,38,39,40].

Recent epidemiologic studies indicated that lung cancer remains one of the most fatal malignancies, despite remarkable improvements that have been made in medical and surgical approaches. Indeed, shortages of highly sensitive screening tests, delays in early screening, and high probabilities of drug resistance and chemoresistance have resulted in increased risks of metastasis and relapse, as well as a meager survival rate for NSCLC patients. Therefore, identifying specific key molecular pathways and highly sensitive biomarkers for NSCLC is urgently needed to formulate effective treatments through personalized medicine [41]. 

## 2. Materials and Methods

### 2.1. Oncomine Gene Analysis for Expression Levels of FKBP Family Members in LUAD

The Oncomine platform (http://www.oncomine.org/, accessed on 22 November 2021) is an online cancer microarray bioinformatics database established to display transcriptional levels of target cancer and normal specimens from 715 datasets [42,43,44]. In this research, we analyzed individual mRNA expression levels of *FKBP* family members in various types of cancer via Oncomine with the setting of *p* < 0.01, fold change of 1.5, and gene rank in the top 10%.

### 2.2. Gene Expression Profiling Interactive Analysis (GEPIA) 2 Analysis for Clinicpathological States of FKBP Family Members

GEPIA 2 (http://gepia2.cancer-pku.cn/#index/, accessed on 22 November 2021) is a beneficial bioinformatics database that offers precise analyses of transcriptional levels of mRNA expressions for 8587 normal tissues and 9736 cancer samples from The Genotype-Tissue Expression (GTEx) project (https://www.gtexportal.org/home/, accessed on 22 November 2021) and The Cancer Genome Atlas (TCGA) (https://tcga-data.nci.nih.gov/tcga/, accessed on 22 November 2021) [43,45,46]. GEPIA 2 supplies major functions including gene expression analyses, gene correlation analyses, survival analyses, similar gene predictions, and dimensionality reduction analyses. In the present study, GEPIA 2 was used to explore gene expressions in different stages and for normal/cancer tissue comparisons.

### 2.3. Kaplan-Meier (KM) Plotter Survival Assessment of FKBP Gene Family Members

The KM plotter (http://kmplot.com/analysis/, accessed on 30 November 2021) is a visual bioinformatics database containing up to 54,000 genes in 21 cancer types including lung, ovarian, breast, and gastric cancers [47]. This public database can be used to conduct meta-analyses with TCGA, Gene Expression Omnibus (GEO) (https://www.ncbi.nlm.nih.gov/geo/, accessed on 30 November 2021), and European Genome-phenome Archive (EGA) (https://ega-archive.org/, accessed on 30 November 2021) [48]. We adopted a pan-cancer platform to analyze the prognostic merits of transcriptional levels of individual *FKBP* family members in LUAD patients (*n* = 513) for OS and relapse-free survival (RFS) with the KM plotter choosing median values, a *p* log rank of <0.05, and hazard ratios (HRs) of >1.

### 2.4. cBioPortal Analysis of Genetic Alterations of FKBP Family Members in LUAD

cBioPortal (http://cbioportal.org/, accessed on 30 November 2021) is an online resource that integrates several cancer-related databases to analyze genetic alterations, DNA methylation, copy number changes, etc. [49,50,51] from more than 5000 cancer specimens in 20 cancer studies [44]. In this study, we explored genetic alterations of the *FKBP* gene family in LUAD with 503 complete samples from TCGA in cBioPortal.

### 2.5. Gene MANIA Was Used to Build Gene-Gene Interactions (GGIs) and Explore Their Functions

Gene MANIA (http://www.genemania.org/, accessed on 4 December 2021) is a versatile tool for predicting gene functions, analyzing gene lists, and recognizing the most interrelated genes such as *Homo sapiens,* based on more than 800 connections [52,53]. We examined GGI networks and functions of *FKBP* family numbers by Gene MANIA.

### 2.6. STRING Analysis of the FKBP Gene Family and Other Associations of Expressed Proteins

The purpose of the STRING informatics tool (https://string-db.org/, accessed on 4 December 2021) is to gather and integrate accessible sources of protein-protein interactions (PPIs) and conduct computational forecasts. The aim of this tool is to achieve a comprehensive analysis [54]. The newest version of STRING (11.5) for organisms, renewed on 12 August 2021, is nearly triple the size of the older version (11.0b) and covers 1,409,467,592,464 proteins and 20,052,394,042 interactions.

### 2.7. Database for Annotation, Visualization and Integrated Discovery (DAVID) and MetaCore Analysis of Coexpressions of FKBP Family Members

DAVID 6.8 (https://david.ncifcrf.gov/, accessed on 4 December 2021) provides a platform to facilitate analysis of gene lists of interest [55], and data visualization through online platform (http://www.bioinformatics.com.cn/srplot, accessed on 4 December 2021). These platform consists of the Kyoto Encyclopedia of Genes and Genomes (KEGG) and gene ontology (GO) and is composed of molecular functions (MFs), biological processes (BPs), and cellular components (CCs) [56]. The goal of KEGG is to assign biological functions to genes and genomes [57], while GO offers information about gene products, processes, and functions [58]. Together with the MetaCore platform, we mapped the intersection between these two sets of data in terms of related pathways and involved networks. A *p* value of <0.05 was considered significant, as previously described.

### 2.8. Tumor Immune Estimation Resource (TIMER) 1.0 Comprehensive Investigation of Components of Immune Cell Infiltration of FKBP Gene Family Members in LUAD

TIMER 1.0 (http://timer.comp-genomics.org/, accessed on 4 December 2021) is a convenient server for the analysis and visualization of associations of target genes and related immune cells between tumor and normal samples from 10,897 samples in 32 cancer types [59,60]. In this research, we analyzed correlations of different *FKBP* family members in LUAD with the enrichment of immune cell infiltrates, including B cells, cluster of differentiation 8-positive (CD8^+^) T cells, CD4^+^ T cells, macrophages, neutrophils, and dendritic cells (DCs).

### 2.9. Statistical Analysis

We utilized TCGA Pan-Cancer Atlas, a dataset from cBioPortal, to obtain patient data and query the effects of the expressions of different *FKBP* family members on OS. For the survival analysis, a KM plotter was applied, with all default settings, and RFS was preferred, with automatic cutoff values and J best probe set. All possible cutoff values between the lower and upper quartiles were determined, and the best presenting threshold was subsequently used as the cutoff [51]. A log-rank *p* value of <0.05 was considered statistically significant.

## 3. Results

**Figure 1:**
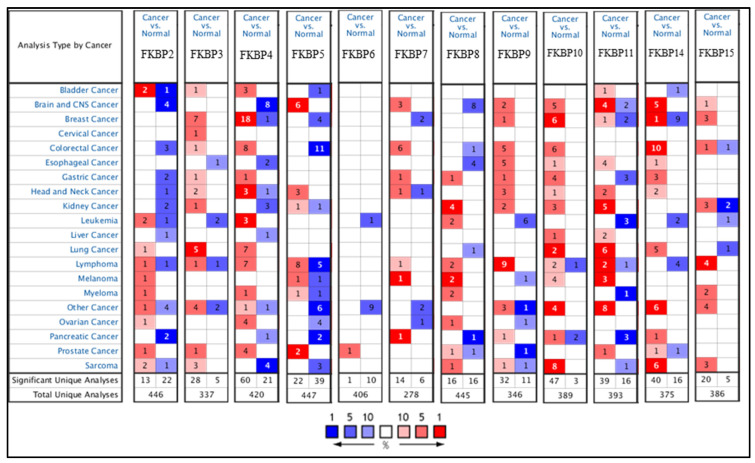
The mRNA expression levels of *FKBP* family members in various cancer types analyzed using the Oncomine database. Transcriptional levels of *FKBP2*, *FKBP3*, *FKBP4*, *FKBP10*, *FKBP11*, and *FKBP14* were higher in lung cancer samples than in normal samples. Red and blue cells, respectively, represent statistically upregulated and downregulated mRNA expression levels of *FKBP* family members. The threshold was set to a fold change of *p* < 0.05; fold change > 1.5 *p* = 0.05; gene rank top 10%.

Transcriptional levels of *FKBP* family members were linked to various cancers due to abnormal expressions, but no publications have mentioned the connection between *FKBP* family genes and LUAD. In the present study, we compared levels of *FKBP* transcripts in different tumor tissues and normal samples by utilizing an Oncomine analysis (Figure 1). Findings indicated that mRNA levels of *FKBP2, FKBP3, FKBP4, FKBP10, FKBP11*, and *FKBP14* were overexpressed in LUAD tissues compared to healthy tissues. In the datasets from Stearmen et al. [61], *FKBP2* expression was elevated in LUAD patients, with a fold change of 1.520 (*p* = 2.94 × 10^−5^) (Supplementary Table S1). In three datasets, transcriptional levels of *FKBP3* in tumor tissues were higher than those in normal samples. In the LUAD datasets of Su et al. [62], *FKBP3* mRNA was significantly overexpressed in LUAD tissues, with a fold change of 1.761 (*p* = 8.06 × 10^−9^), and in addition, datasets from Landi et al. [63] revealed that the increase in transcriptional levels of *FKBP3* in LUAD was invalid, with a fold change of 1.724 (*p* = 1.00 × 10^−16^). Additionally, Hou et al.’s datasets [64] showed upregulated fold changes of 1.600 (*p* = 9.13 × 10^−12^), 1.783 (*p* = 3.84 × 10^−12^), and 1.810 (*p* = 6.73 × 10^−7^) of *FKBP3* in LUAD, SCLC, and LCLC, respectively. In LUAD datasets of Stearman et al. [61] and Beer et al. [65], compared to normal tissues, *FKBP4* mRNA was obviously higher with fold changes of 1.715 (*p* = 2.02 × 10^−7^) and 1.523 (*p* = 6.99 × 10^−6^), respectively. The SCLC datasets of Wachi et al. [66] stated that *FKBP4* was overexpressed by a fold change of 2.085 (*p* = 3.19 × 10^−4^). In Hou et al.’s datasets [64], *FKBP4* was also discovered to be upregulated in LCLC, SCLC, and LUAD with respective fold changes of 3.607 (*p* = 3.84 × 10^−8^), 2.765 (*p* = 9.11 × 10^−15^), and 2.082 (*p* = 3.46 × 10^−9^). Transcription levels of *FKBP4* were found to be increased in SCLC patients in Bhattacharjee et al.’s datasets [67], with a fold change of 3.530 (*p* = 0.002). In Garber et al.’s datasets [68], *FKBP10* expression was also reported to be higher in LCLC and SCLC, with respective fold changes of 4.332 (*p* = 2.17 × 10^−4^) and 2.417 (*p* = 0.003). *FKBP11* was overexpressed in LUAD, with fold changes of 2.826 (*p* = 2.20 × 10^−6^), 2.031 (*p* = 2.81 × 10^−7^), 2.114 (*p* = 1.02 ×10 ^−13^), 2.340 (*p* = 1.82 × 10^−9^), 2.002 (*p* = 2.05 × 10^−10^), and 1.740 (*p* = 2.53 × 10^−9^) in the datasets of Garber et al. [68], Su et al. [62], Landi et al. [63], Hou et al. [64], Okayama et al. [69], and Selatmat et al. [70], respectively. Transcriptional levels of *FKBP14* were upregulated in LUAD, with fold changes of 1.948 (*p* = 1.37 × 10^−6^), 1.781 (*p* = 0.002), 1.722 (*p* = 1.33 × 10^−8^), and 3.691 (*p* = 1.27 × 10^−10^) in datasets of Su et al. [62], Garber et al. [68], Hou et al. [64], and Selamat et al. [70], respectively. In SCLC, *FKBP14* expression was elevated as was proven by Hou et al.’s [64] datasets. Furthermore, expressions of *FKBP8* and *FKBP15* were lower in LUAD in Bhattacharjee et al.’s datasets [67], with a fold change of −4.273 (*p* = 6.80 × 10^−5^), and a lower LCLC in Hou et al.’s datasets [64], with a fold change of −1.839 (*p* = 4.87 × 10^−13^). However, mRNA expression levels of *FKBP5, FKBP6, FKBP7,* and *FKBP9* exhibited no significant differences between lung cancer tissues and healthy samples (Supplementary Table S1). In addition, we also analyzed the expressions of *FKBP* family members in cancer and normal tissues and pathological features in LUAD using GEPIA 2 (Supplementary Figure S1).

### 3.1. Survival Analysis and Prognostic Values of FKBP Family Members in LUAD

**Figure 2:**
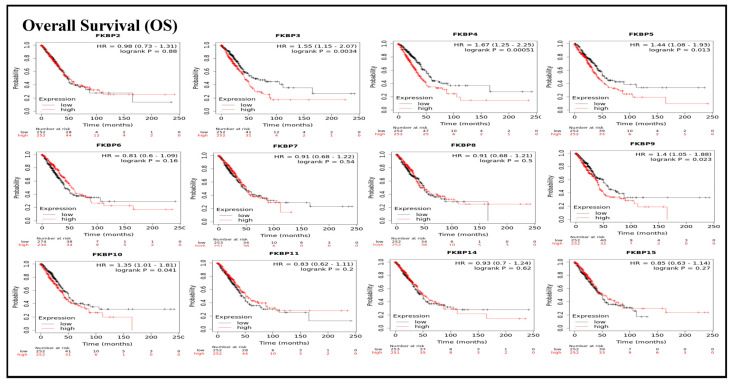
Prognoses of mRNA expressions of *FKBP* gene family members in lung adenocarcinoma (LUAD) patients in the overall survival (OS) analysis using the KM plotter database. Low expression levels of *FKBP3*, *FKBP4*, *FKBP5*, *FKBP9*, and *FKBP10* were significant relative to the higher OS values. The setting for filtering LUAD patients was the median level.

**Figure 3:**
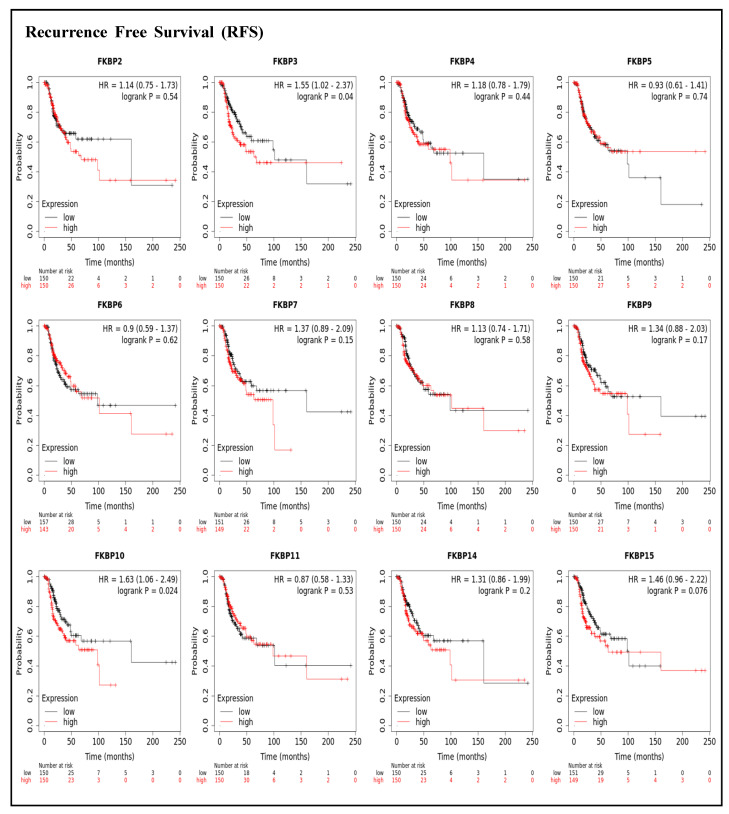
Prognoses of mRNA expression levels of *FKBP* gene family members in lung adenocarcinoma (LUAD) patients in terms of a relapse-free survival (RFS) analysis using the KM plotter database. Low expression levels of *FKBP3* and *FKBP10* were significantly related to greater RFS values. The setting for filtering LUAD patients was the median level.

To assess associations between distinct transcriptional levels of *FKBP* family members and survival rates of LUAD, we used the KM plotter database, and preferentially analyzed OS (Figure 2). Outcomes revealed that five members of the *FKBP* gene family had correlations with poor prognoses among LUAD patients in the OS analysis including *FKBP3* (*p* = 0.0034; hazard ratio (HR) = 1.55), *FKBP4* (*p* = 0.00051; HR = 1.67), *FKBP5* (*p* = 0.013; HR = 1.44), *FKBP9* (*p* = 0.023; HR = 1.4), and *FKBP10* (*p* = 0.041; HR = 1.35). However, transcriptional levels of *FKBP2, FKBP6, FKBP7, FKBP8 FKBP11, FKBP14*, and *FKBP15* were not linked to the OS of LUAD patients. mRNA expressions of *FKBP* family memberss in relation to RFS were also analyzed (Figure 3). Low transcriptional levels of *FKBP3* (*p* = 0.04; HR = 1.55) and *FKBP10* (*p* = 0.024; HR = 1.63) in LUAD patients were correlated with a longer RFS, while the remaining *FKBP* family members were not related to RFS in LUAD samples (Supplementary Table S2).

### 3.2. Genetic Alteration Analysis of FKBP Family Members in LUAD

**Figure 4:**
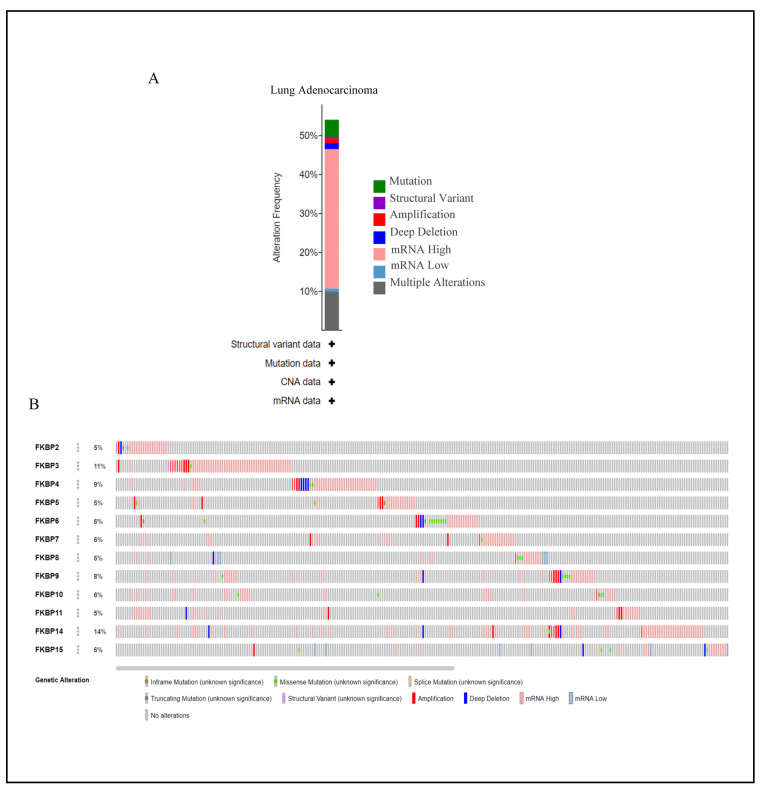
Analysis of genetic alterations of *FKBP* family numbers in lung adenocarcinoma (LUAD) samples using the CBioPortal database. (**A**) Summary of genetic alterations in distinctive *FKBP* gene family members in LUAD. (**B**) OncoPrint dataset showing the *FKBP* family numbers showing alterations of individual *FKBPs* on queried genes. The horizontal axis represents each LUAD patient from the TCGA database, and the rate of genetic alterations in *FKBP* family members ranged from 5% to 14%.

Mutations in *FKBP* family numbers had large impacts on FK506 binding and its enzymatic functions [71]. Thus, we conducted a visual analysis to gain insights into genetic alterations of the *FKBP* gene family alongside the cBioPortal bioinformatics tool from TCGA. Results revealed that *FKBP* family members were altered in 272 instances among 503 LUAD patients, and these alterations included mutations, structural variants, amplifications, deep deletions, mRNA high, mRNA low, and multiple alterations based on the dataset (Figure 4A). The rate of genetic alterations in *FKBP* family members ranged from 5% to 14% (*FKBP2, FKBP5, FKBP8*, and *FKBP11*: 5%; *FKBP6, FKBP7, FKBP10*, and *FKBP15*: 6%; *FKBP9*: 8%; *FKBP4*: 9%; *FKBP3*: 11%; and *FKBP14*: 14%) (Figure 4B).

### 3.3. Analysis of GGIs and PPIs and Coexpression of Pathway Abundance of the FKBP Gene Family

Due to genetic diversity, GGIs have impacts on gene functions, relative pathways, and even the development of target drugs [72]. In this study, we analyzed GGI networks of *FKBP* family members with neighbor genes via GeneMANIA (Supplementary Figure S2A). Results showed that *HECTD1*, *TTC6*, and other correlated genes were intensely linked with *FKBP* family members in shared protein domains (55.30%); coexpression (31.22%); physical interactions (13.16%); predictions (0.23%); co-localization (0.08%); and genetic interactions (0.02%); and functions including drug binding, cis-trans isomerase activity, protein folding, peptidyl-proline medication, isomerase activity, negative regulation of calcium ion transport into the cytosol, and positive regulation of the sequestration of calcium ions. Additionally, because cellular life is based on a complicated network of functional influences among biomolecules [73], we also evaluated the PPIs of *FKBP* family members in *Homo sapiens* using the STRING database (Supplementary Figure S2B). 

The current data revealed that mRNA levels of *FKBP2*, *FKBP3*, *FKBP4*, *FKBP10*, *FKBP11*, and *FKBP14* were overexpressed in LUAD, and *FKBP10* had connections to poor prognoses among LUAD patients in an OS analysis. Based on the above results, we selected *FKBP10* to further conduct a comprehensive analysis of the downstream pathway and network. Next, to deeply analyze coexpressed genes with *FKBP10*, we downloaded an archive of genes coexpressed with *FKBP10* in LUAD and chose data for the first 1000 small *p* values from cBioPortal before performing DAVID. We analyzed two different aspects. First, GO term enrichment (GOTERM) revealed several *FKBP10*-correlated pathways, including protein binding (*p* = 1.6 × 10^−16^), isomerase activity (*p* = 8.6 × 10^−2^), etc. (Supplementary Figure S3, Supplementary Table S3). GOTERM_BPs described biological events in which these coexpressed genes of *FKBP10* were involved, including mitochondrial electron transport, NADH to ubiquinone, mitochondrial respiratory chain complex I assembly, etc. (Supplementary Figure S4, Supplementary Table S4). The CCs of genes coexpressed with *FKBP10* were also revealed in GOTERM_(CC) (Supplementary Figure S5, Supplementary Table S5). In another aspect of a KEGG analysis, outcomes indicated that there were 325 coexpressed genes (33.8%) in the non-alcoholic fatty liver disease (NAFLD) pathway (Supplementary Figure S6).

**Figure 5:**
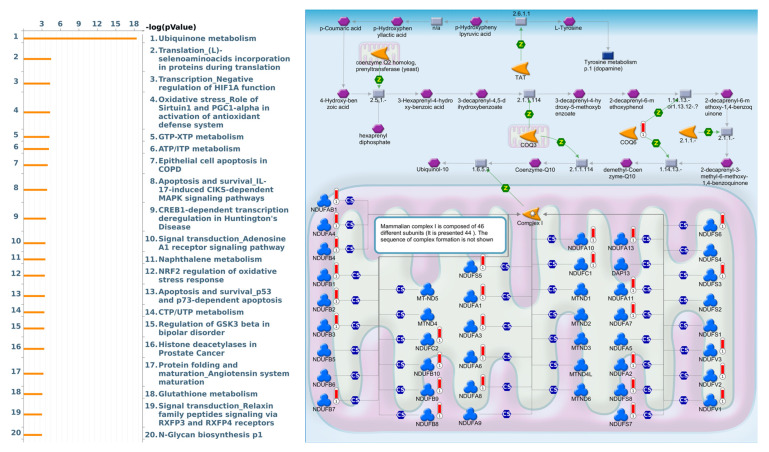
MetaCore pathway analysis of the coexpression gene network of FKBP family members in lung adenocarcinoma (LUAD) patients. The MetaCore pathway analysis of “biological processes” revealed that “Ubiquinone metabolism”-related pathways were correlated with LUAD development.

As a result, annotations of almost all BPs obtained from GeneGo Metacore showed that genes coexpressed with *FKBP10* participated in several networks and cell-cycle-related pathways such as “Ubiquinone metabolism”, “Translation_(L)-selenoaminoacids incorporation in proteins during translation”, “Transcription_Negative regulation of HIF1A function”, “Oxidative stress_Role of Sirtuin1 and PGC1-alpha in activation of antioxidant defense system”, “GTP-XTP metabolism”, “ATP/ITP metabolism”, “Epithelial cell apoptosis in COPD”, “Apoptosis and survival_IL-17-induced CIKS-dependent MAPK signaling pathways”, “CREB1-dependent transcription deregulation in Huntington’s disease”, and “Signal transduction_Adenosine A1 receptor signaling pathway” (Figure 5, Supplementary Table S6).

### 3.4. Levels of Immune Cell Infiltration of Different FKBP Family Members in LUAD Patients

The TME is extremely important for the existence of cancer and is composed of vessels, extracellular matrix (ECM), and various immune cells, which favor tumor invasion, proliferation, and metastasis. Therefore, the occurrence of cancer is closely related to immune cells [74,75]. To fully understand the associations between *FKBP* family members and immune cell infiltration in LUAD, we evaluated the immunological microenvironment using the TIMER database (Supplementary Figure S7). Results indicated that *FKBP2* was negatively correlated with CD8^+^ T cells (*r* = −0.345; *p* = 4.28 × 10^−15^), macrophages (*r* = −0.26; *p* = 5.8 × 10^−9^), neutrophils (*r* = −0.304; *p* = 8.41 × 10^−12^), and DCs (*r* = −0.283; *p* = 2.08 × 10^−10).^ Transcriptional levels of *FKBP3* were negatively associated with B cells (*r* = −0.114; *p* = 1.2 × 10^−2^), CD8^+^ T cells (*r* = −0.112; *p* = 1.33 × 10^−10^), CD4^+^ T cells (*r* = −0.133; *p* = 3.48 × 10^−3^), macrophages (*r* = −0.194; *p* = 1.72 × 10^−5^), neutrophils (*r* = −0.105; *p* = 2.16 × 10^−2^), and DCs (*r* = −0.167; *p* = 2.13 × 10^−4^). *FKBP4* expression showed a positive correlation with purity (*r* = 0.12; *p* = 7.59 × 10^−3^) but negative correlations with B cells (*r* = −0.265; *p* = 3.34 × 10^−9^), CD4^+^ T cells (*r* = −0.151; *p* = 8.51 × 10^−4^), and DCs (*r* = −0.099; *p* = 2.84 × 10^−2^). *FKBP5* had a negative association with purity (*r* = −0.125; *p* = 5.53 × 10^−3^) but positive associations with CD8^+^ T cells (*r* = 0.242; *p* = 6.56 × 10^−8^), macrophages (*r* = 0.194; *p* = 1.65 × 10^−5^), neutrophils (*r* = 0.219; *p* = 1.11 × 10^−6^), and DCs (*r* = 0.231; *p* = 2.4 × 10^−7^). *FKBP6* expression was positively correlated with B cells (*r* = 0.245; *p* = 4.49 × 10^−8^), CD4^+^ T cells (*r* = 0.18; *p* = 6.72 × 10^−5^), macrophages (*r* = 0.129; *p* = 4.34 × 10^−3^), and DCs (*r* = 0.129; *p* = 4.39 × 10^−3^). Correlations of *FKBP7* with immune cells were negative in terms of purity (*r* = −0.102; *p* = 2.4 × 10^−2^) and positive in terms of macrophages (*r* = 0.12; *p* = 7.91 × 10^−3^). *FKBP8* expression was negatively associated with CD8^+^ T cells (*r* = −0.241; *p* = 7.7 × 10^−8^) and positively associated with CD4^+^ T cells (*r* = 0.302; *p* = 1.08 × 10^−11^). *FKBP9* expression exhibited a positive association with neutrophils (*r* = 0.136; *p* = 2.72 × 10^−3^). Transcriptional levels of *FKBP10* showed a negative link with B cells (*r* = −0.1; *p* = 2.8 × 10^−2^). *FKBP11* expression revealed negative associations with purity (*r* = −0.159; *p* = 4.05 × 10^−4^), macrophages (*r* = −0.164; *p* = 2.88 × 10^−4^), neutrophils (*r* = −0.093; *p* = 4.09 × 10^−2^), and DCs (*r* = −0.19; *p* = 2.35 × 10^−5^) and a positive association with B cells (*r* = 0.129; *p* = 4.48 × 10^−3^). *FKBP14* expression was negatively related to purity (*r* = −0.138; *p* = 2.13 × 10^−3^) but positively associated with CD8^+^ T cells (*r* = 0.114; *p* = 1.21 × 10^−2^), macrophages (*r* = 0.14; *p* = 2.01 × 10^−3^), neutrophils (*r* = 0.242; *p* = 7.16 × 10^−8^), and DCs (*r* = 0.181; *p* = 5.81 × 10^−5^). *FKBP15* expression was negatively associated with all cells analyzed including purity (*r* = −0.262; *p* = 3.47 × 10^−9^), and positively associated with B cells (*r* = 0.334; *p* = 4.53 × 10^−14^), CD8^+^ T cells (*r* = 0.204; *p* = 5.64 × 10^−6^), CD4^+^ T cells (*r* = 0.535; *p* = 4.01 × 10^−37^), macrophages (*r* = 0.405; *p* = 1.29 × 10^−2^), neutrophils (*r* = 0.581; *p* = 4.89 × 10^−45^), and DCs (*r* = 0.612; *p* = 1.73 × 10^−51^). These outcomes revealed that the *FKBP* gene family plays important roles in immunological effects.

## 4. Discussion

In this study, we analyzed *FKBP* mRNA expressions, clinical phase IV, survival rates, genetic variants, and coexpressed genes. Moreover, we also determined correlations of infiltration levels of immune cells and *FKBP* gene expressions in LUAD. Although direct evidence still needs to be provided as to the biological functions of *FKBP*, such as cell models or patient tissue samples, to demonstrate the roles of *FKBP* in LUAD, based on our results, we can provide the concept that *FKBP* can potentially be a biomarker in LUAD.

By applying advances in high-throughput screening to cancer transcriptome profiling, alterations in the transcriptome patterns of *FKBP* gene families were found to be significantly associated with several types of malignancies [76,77,78,79,80]. *FKBP* gene expressions were found to be involved in tumor multi-stage progression along with other tumor-related factors. According to various database analyses, *FKBP3, FKBP4*, and *FKBP10* were closely associated with LUAD. Previous studies determined that *FKBP3* is a crucial oncogene in distinct cancers. The combination of *FKBP3* and *HDAC2* was related to oxaliplatin resistance in colorectal cancer via the PTEN/AKT pathway [18]. Downregulation of *FKBP3* suppressed breast cancer [81], and *FKBP3* expression was associated with poor survival in LUAD [82,83,84]. Furthermore, *FKBP4* was reported to be related to breast cancer [20,85], colorectal cancer [22], prostate cancer [86], and lung cancer [21,87]. *FKBP10* was connected with gastric cancer [88], stomach adenocarcinomas [89], papillary thyroid cancer [90], and lung cancer.

Our data were found to be consistent with those in previous research, as current findings indicated that mRNA levels of *FKBP2, FKBP3, FKBP4, FKBP10, FKBP11*, and *FKBP14* were overexpressed in LUAD compared to healthy tissues. The OS analytical results revealed that five members of the *FKBP* gene family, viz., *FKBP3, FKBP4, FKBP5, FKBP9*, and *FKBP10,* had connections with poor prognoses among LUAD patients.

Based on the above results, we selected *FKBP10* to further conduct a comprehensive analysis of the downstream pathway and network. Therefore, we explored the characteristics and functions of *FKBP10* in more detail and discovered that *FKBP10*, also called *FKBP65* (65-kDa), possesses four cytosolic PPI activities [88,91]. Moreover, downregulation of *FKBP10* with collagen VI increased the formation of primary human lung fibroblasts (phLFs) [88]. Additionally, downregulation of *FKBP10* suppressed tumorsphere formation by regulating protein translation [92]. Genes coexpressed with *FKBP10* in TCGA LUAD were subsequently used to perform a pathway analysis. Through the DAVID analysis, we found *FKBP10* to be involved in mitochondrial electron transport, NADH to ubiquinone and mitochondrial respiratory chain complex I assembly, etc. The Metacore pathway analysis also indicated that *FKBP10* was involved in “Ubiquinone metabolism”, “Translation_(L)-selenoaminoacids incorporation in proteins during translation”, and “Transcription_Negative regulation of HIF1A function”.

In recent years, cancer immunotherapy, a novel strategy that aims to activate and boost the immune system to directly recognize and eliminate tumor cells, has undergone tremendous developments, and is now regarded as a promising cancer treatment [93,94,95]. Increasing evidence indicates that the immunosuppressive environment mediated by tumor-infiltrating immune cells (TICs), such as regulatory T (Treg) cells and tumor-associated macrophages (TAMs), hinders the delivery of immunotherapies in LUAD. Our data suggested that TICs are strongly correlated with *FKBP* expressions. Therefore, this analysis of the tumor immune microenvironment could help develop clinical immunotherapies and provide accurate personalized treatment plans for patients.

## 5. Conclusions

Previous research had not fully explored the roles of *FKBP* family genes in LUAD. Consequently, this study represents the first work that specifically examined the roles of *FKBP* members in this disease, prior to providing a more-extensive and -incisive understanding of the potential therapeutic and prognostic value for LUAD patients.

## Data Availability

The datasets used and analyzed during the current study are available from the corresponding author upon reasonable request.

## Supplementary Materials

The following supporting information can be downloaded at: https://www.mdpi.com/article/10.3390/jpm13010049/s1, Supplementary Figure S1: The transcriptional levels of FKBP family member in LDAC and relationship between clinical stages in development of LUAD analyzed by GEPIA 2 database. (A) mRNA expression of FKBP5 in LUAD was greater than normal samples. The q-value cut-off was set to 0.01. (B) The stage plot indicated FKBP4 and FKBP5 were correlated with clinical stages in LUAD known by the height of the white dot in the four stages; Supplementary Figure S2: Analysis of association and abundance of FKBP family members (A) Connections between different FKBP family members and neighbor genes in Homo sapiens by the GeneMANIA database. Each node stands for an individual gene, and the size of the node represents the intensity of the gene-gene interaction (GGI). (B) Protein-protein interaction (PPI) associations between expressed FKBP10 and predicated proteins by the STRING database. The setting for maximum number of interactors to show was no more than 50 interactors; Supplementary Figure S3. Exploration of GOTERM_MF pathways for the coexpression of FKBP10 in lung adenocarcinoma (LUAD) by combining cBioPortal and the DAVID database; Supplementary Figure S4: Exploration of GOTERM_BP pathways for the coexpression of FKBP10 in lung adenocarcinoma (LUAD) by combining cBioPortal and the DAVID database; Supplementary Figure S5. Exploration of GOTERM_CC pathways for the coexpression of FKBP10 in lung adenocarcinoma (LUAD) by combining cBioPortal and the DAVID database; Supplementary Figure S6. Exploration of KEGG pathways for the coexpression of FKBP10 in lung adenocarcinoma (LUAD) by combining cBioPortal and the DAVID database. * Listed genes shown in the diagram are marked by a red star; Supplementary Figure S7. Associations between FKBP family members and immune infiltration consisting of purity, B cells, cluster of differentiation-positive (CD8^+^) T cells, CD4^+^ T cells, macrophages, neutrophils, and dendritic cells from TCGA in lung adenocarcinoma (LUAD) by the TIMER database. * Means a partial correlation (r), and *p* < 0.05 indicates a significant difference; Supplementary Table S1: Significant changes in expressions of FKBP family members between different types of lung cancer and normal tissue; Supplementary Table S2: Associations of prognoses with transcription mRNA levels of FKBP family members in patients with lung cancer; Supplementary Table S3: Gene Ontology term enrichment (GOTERM)_ revealed several FKBP10 correlated pathways and molecular function; Supplementary Table S4: Gene Ontology term enrichment (GOTERM)_ revealed several FKBPs correlated pathways and biological process; Supplementary Table S5: Gene Ontology term enrichment (GOTERM)_ revealed several FKBP10 correlated pathways and cellular components; Supplementary Table S6: GeneGo Metacore showed that the co-expressed genes of FKBP10 participated in several networks.
