# Supplementary material for: Penetrating Exploration of Prognostic Correlations of the FKBP Gene Family with Lung Adenocarcinoma
Source: J Pers Med. 2022 Dec 26;13(1):49. doi: 10.3390/jpm13010049 (PMC9862762; doi:10.3390/jpm13010049)
Supplement: Supplementary file 1 [file jpm-13-00049-s001.zip › jpm-2053681-supplementary.pdf]

SUPPLEMENTARY MATERIAL

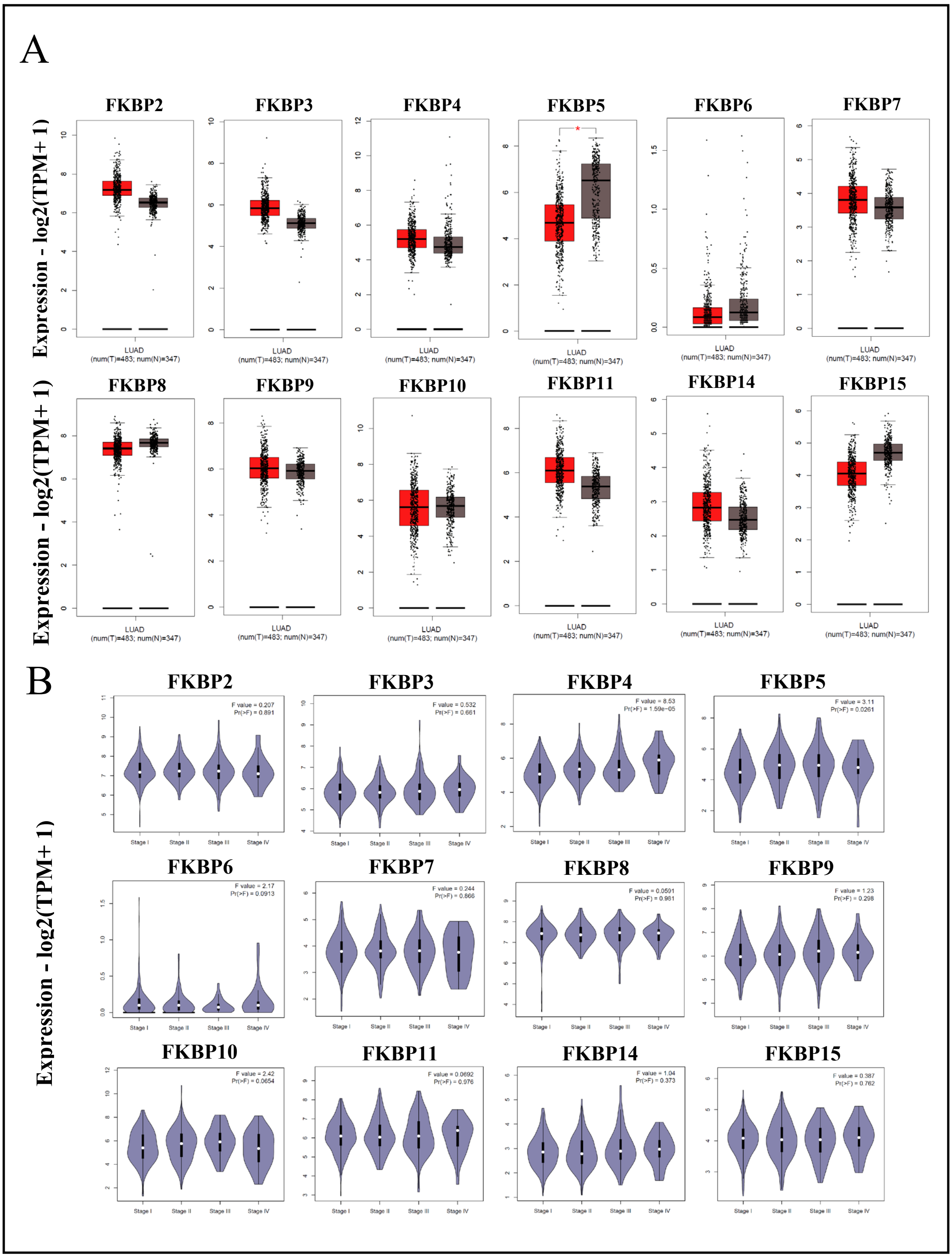

Supplementary Figure S1: The transcriptional levels of *FKBP* family member in LDAC and relationship between clinical stages in development of LUAD analyzed by GEPIA 2 database. (A) mRNA expression of *FKBP5* in LUAD was greater than normal samples. The q-value cut-off was set to 0.01. (B) The stage plot indicated *FKBP4* and *FKBP5* were correlated with clinical stages in LUAD known by the height of the white dot in the four stages.

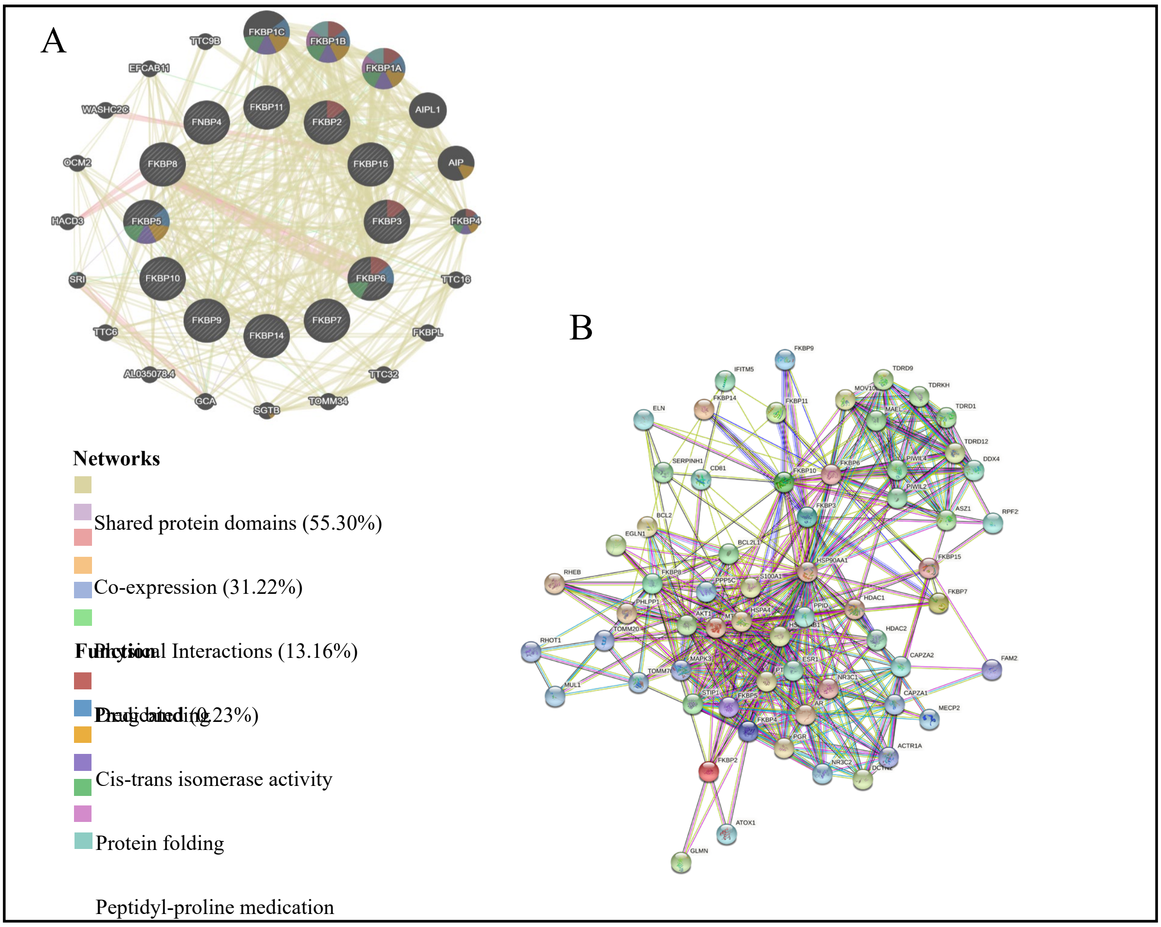

Supplementary Figure S2:. Analysis of association and abundance of *FKBP* family members (A) Connections between different *FKBP* family members and neighbor genes in *Homo sapiens* by the GeneMANIA database. Each node stands for an individual gene, and the size of the node represents the intensity of the gene-gene interaction (GGI). (B) Protein-protein interaction (PPI) associations between expressed *FKBPs* and predicated proteins by the STRING database. The setting for maximum number of interactors to show was no more than 50 interactors.

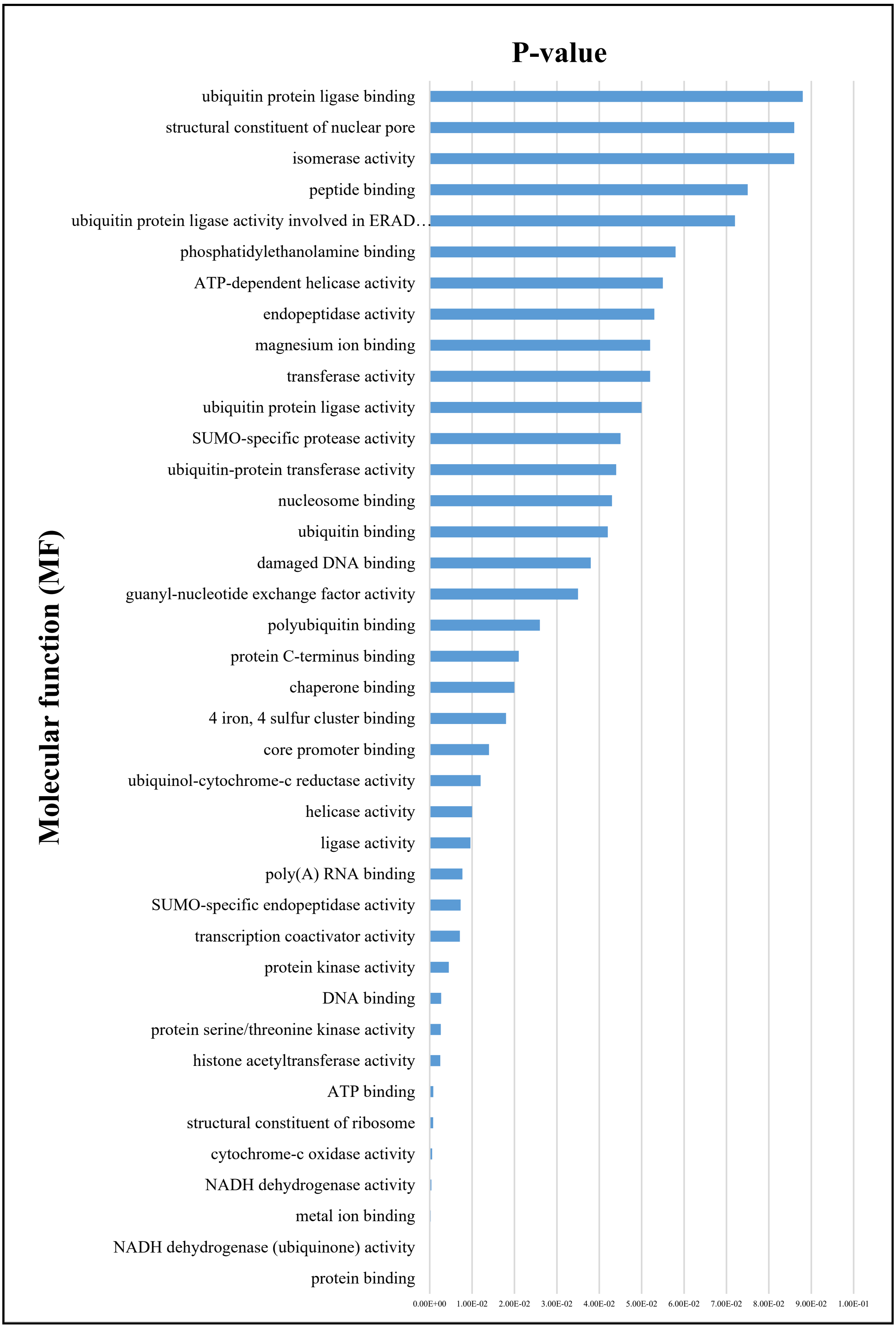

Supplementary Figure S3. Exploration of GOTERM_MF pathways for the coexpression of *FKBP* family members in lung adenocarcinoma (LUAD) by combining cBioPortal and the DAVID database.

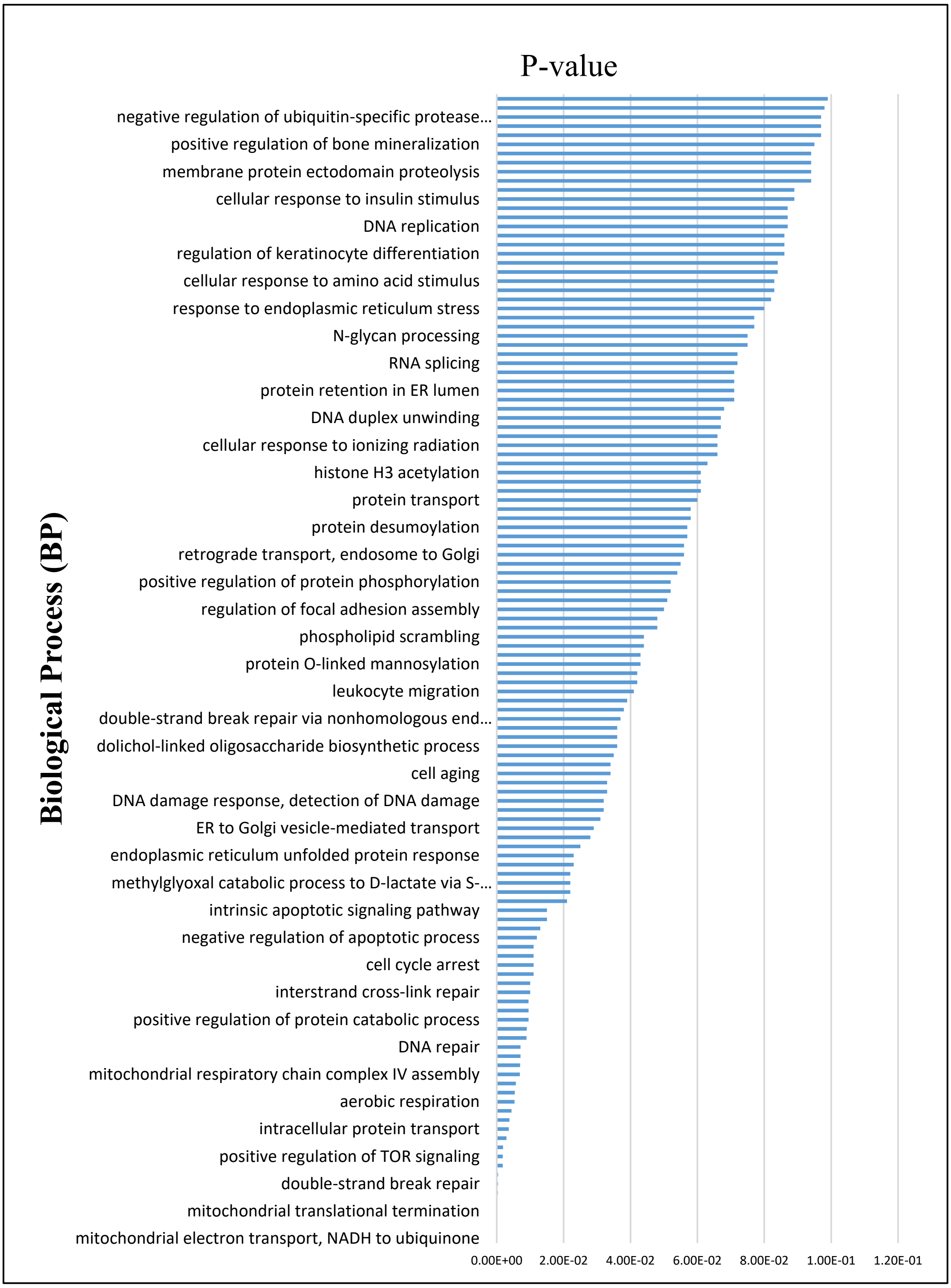

Supplementary Figure S4: Exploration of GOTERM_BP pathways for the coexpression of *FKBP* family members in lung adenocarcinoma (LUAD) by combining cBioPortal and the DAVID database.

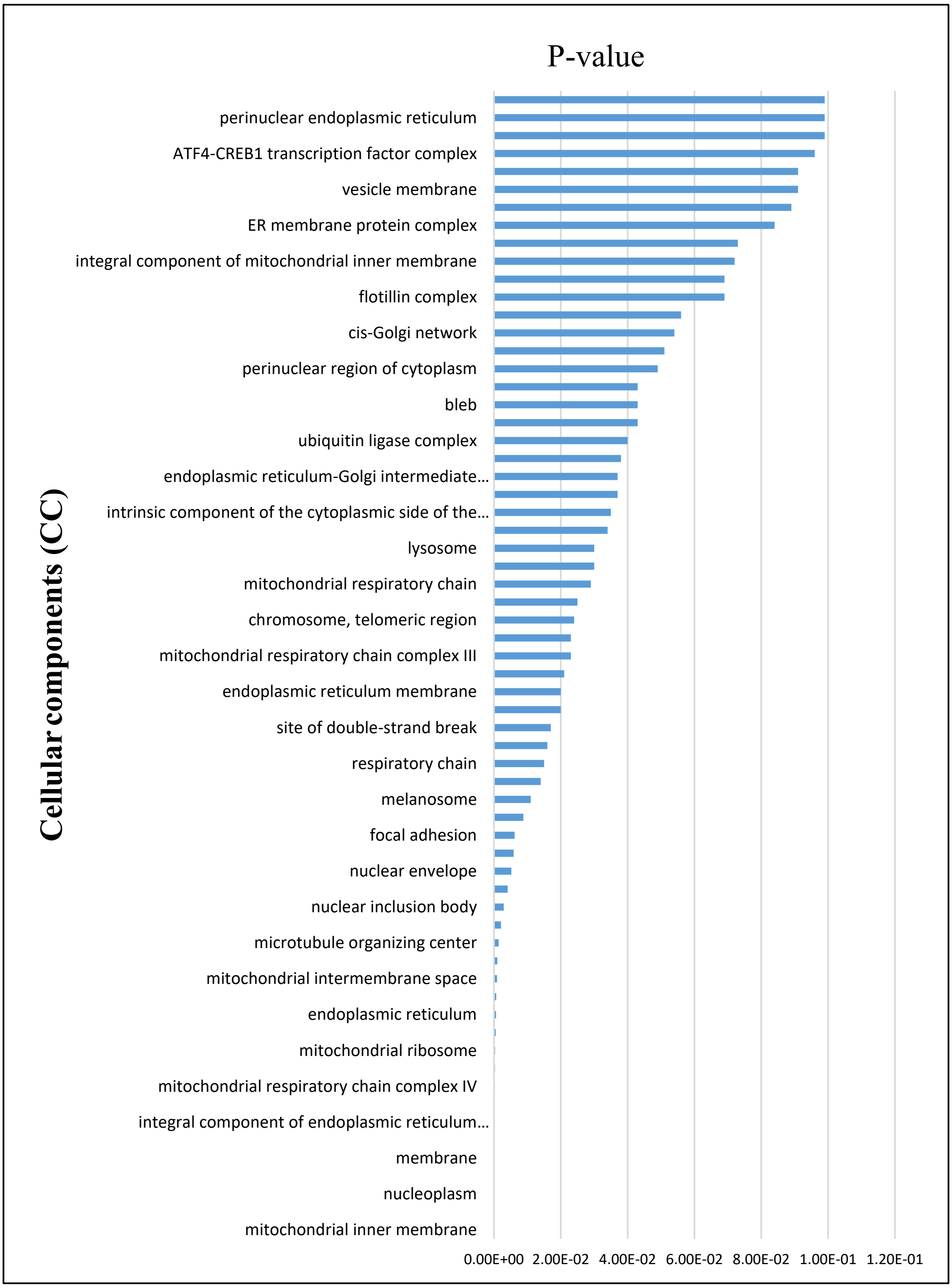

Supplementary Figure S5. Exploration of GOTERM_CC pathways for the coexpression of *FKBP* family members in lung adenocarcinoma (LUAD) by combining cBioPortal and the DAVID database.

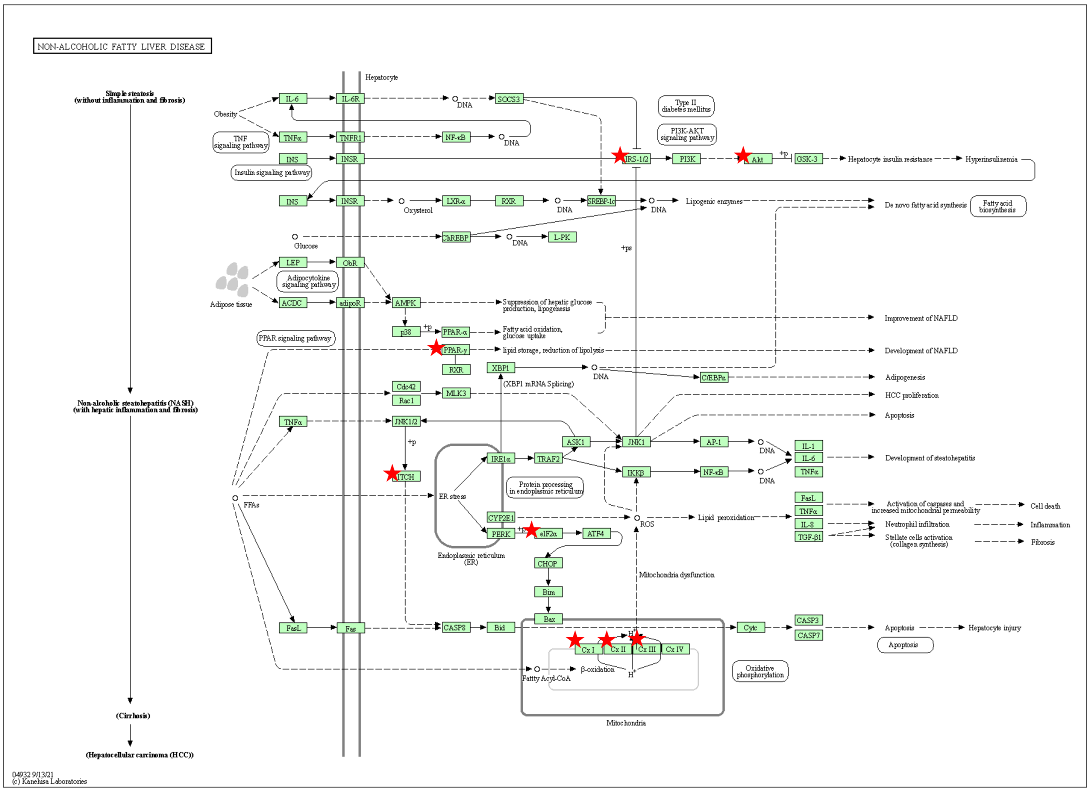

Supplementary Figure S6. Exploration of KEGG pathways for the coexpression of *FKBP* family members in lung adenocarcinoma (LUAD) by combining cBioPortal and the DAVID database. * Listed genes shown in the diagram are marked by a red star.

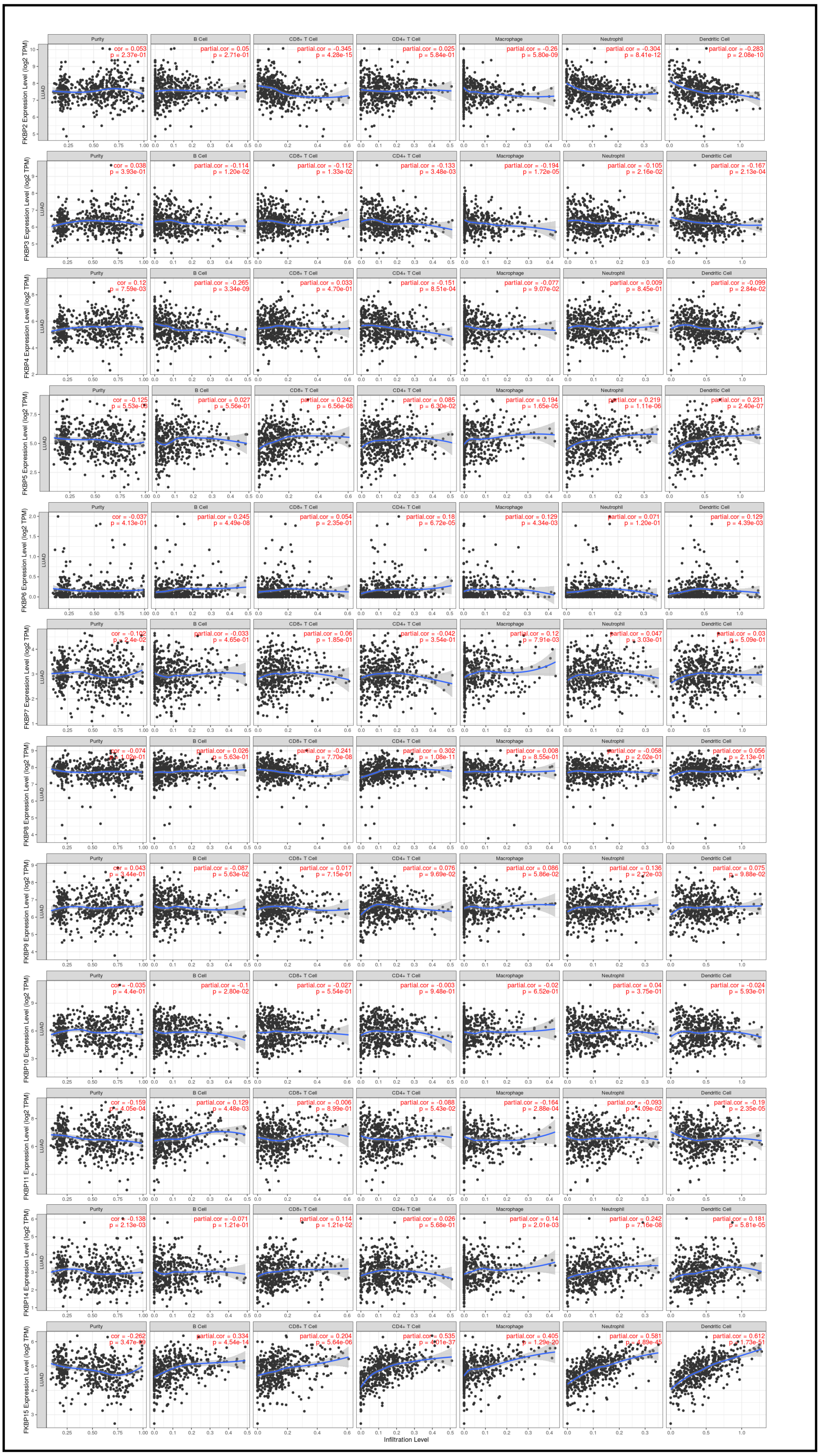

Supplementary Figure S7. Associations between *FKBP* family members and immune infiltration consisting of purity, B cells, cluster of differentiation-positive ($CD8^{+}$) T cells, $CD4^{+}$ T cells, macrophages, neutrophils, and dendritic

cells from TCGA in lung adenocarcinoma (LUAD) by the TIMER database. * Means a partial correlation (*r*), and $p<0.05$ indicates a significant difference.

Supplementary Table S1: Significant changes in expressions of *FKBP* family members between different types of lung cancer and normal tissue

Types of LUAD *vs.* normal		Fold change	t-test	*P*-value
FKBP2	Lung Adenocarcinoma	1.520	4.572	2.94E-5
FKBP3	Lung Adenocarcinoma	1.761	6.692	8.06E-9
	Lung Adenocarcinoma	1.724	9.847	1.00E-16
	Lung Adenocarcinoma	1.600	8.210	9.13E-12
	Squamous Cell Lung Carcinoma	1.783	9.901	3.84E-12
	Large Cell Lung Carcinoma	1.810	6.697	6.73E-7
FKBP4	Lung Adenocarcinoma	1.715	6.896	2.02E-7
	Squamous Cell Lung Carcinoma	2.085	8.045	3.19E-4
	Lung Adenocarcinoma	1.523	5.960	6.99E-6
	Large Cell Lung Carcinoma	3.607	8.145	3.84E-8
	Squamous Cell Lung Carcinoma	2.765	12.638	9.11E-15
	Lung Adenocarcinoma	2.082	6.794	3.46E-9
	Squamous Cell Lung Carcinoma	3.530	3.085	0.002
FKBP5	NA	NA	NA	NA
FKBP6	NA	NA	NA	NA
FKBP7	NA	NA	NA	NA
FKBP8	Lung Adenocarcinoma	-4.273	-4.453	6.80E-5
FKBP9	NA	NA	NA	NA
FKBP10	Large Cell Lung Carcinoma	4.332	6.254	2.17E-4
	Squamous Cell Lung Carcinoma	2.417	3.383	0.003
FKBP11	Lung Adenocarcinoma	2.826	6.753	2.20E-6
	Lung Adenocarcinoma	2.031	5.891	2.81E-7
	Lung Adenocarcinoma	2.114	8.426	1.02E-13
	Lung Adenocarcinoma	2.340	6.600	1.82E-9
	Lung Adenocarcinoma	2.002	8.730	2.05E-10
	Lung Adenocarcinoma	1.740	6.329	2.53E-9
FKBP14	Lung Adenocarcinoma	1.948	5.332	1.37E-6
	Lung Adenocarcinoma	1.781	3.715	0.002
	Lung Adenocarcinoma	1.722	6.292	1.33E-8
	Squamous Cell Lung Carcinoma	2.091	6.977	2.06E-8
	Lung Adenocarcinoma	3.691	7.017	1.27E-10
FKBP15	Large Cell Lung Carcinoma	-1.839	-12.249	4.87E-13

Supplementary Table S2: Associations of prognoses with transcription mRNA levels of *FKBP* family members in patients with lung cancer

	KM Plotter(Logrank P) ; HR			
	OS		RFS	
FKBP2	0.88	0.98 (0.73-1.31)	0.54	1.14 (0.75-1.73)
FKBP3	0.0034	1.55 (1.15-2.07)	0.04	1.55 (1.02-2.37)
FKBP4	0.00051	1.67 (1.25-2.25)	0.44	1.18 (0.78-1.79)
FKBP5	0.013	1.44 (1.08-1.93)	0.74	0.93 (0.61-1.41)
FKBP6	0.16	0.81 (0.6-1.09)	0.62	0.9 (0.59-1.37)
FKBP7	0.54	0.91 (0.68-1.22)	0.15	1.37 (0.89-2.09)
FKBP8	0.5	0.91 (0.68-1.21)	0.58	1.13 (0.74-1.71)
FKBP9	0.023	1.4 (1.05-1.88)	0.17	1.34 (0.88-2.03)
FKBP10	0.041	1.35 (1.01-1.81)	0.024	1.63 (1.06-2.49)
FKBP11	0.2	0.83 (0.62-1.11)	0.53	0.87 (0.58-1.33)
FKBP14	0.62	0.93 (0.7-1.24)	0.2	1.31 (0.86-1.99)
FKBP15	0.27	0.85 (0.63-1.14)	0.076	1.46 (0.96-2.22)

Supplementary Table S3: Gene Ontology term enrichment (GOTERM)_ revealed several *FKBPs* correlated pathways and molecular function

GO	Category	Term	Count	%	Log10(*P*)	Log10(q)
Molecular Function	GO Molecular Function	protein binding	558	58.0	1.6E-16	1.5E-13
Molecular Function	GO Molecular Function	NADH dehydrogenase (ubiquinone) activity	17	1.8	7.1E-10	3.2E-7
Molecular Function	GO Molecular Function	metal ion binding	139	14.4	2.5E-4	7.5E-2
Molecular Function	GO Molecular Function	NADH dehydrogenase activity	5	0.5	3.8E-4	8.5E-2
Molecular Function	GO Molecular Function	cytochrome-c oxidase activity	8	0.8	5.8E-4	1.0E-1
Molecular Function	GO Molecular Function	structural constituent of ribosome	24	2.5	8.2E-4	1.1E-1
Molecular Function	GO Molecular Function	ATP binding	103	10.7	8.5E-4	1.1E-1
Molecular Function	GO Molecular Function	histone acetyltransferase activity	9	0.9	2.5E-3	2.4E-1
Molecular Function	GO Molecular Function	protein serine/threonine kinase activity	33	3.4	2.6E-3	2.4E-1
Molecular Function	GO Molecular Function	DNA binding	110	11.4	2.7E-3	2.4E-1
Molecular Function	GO Molecular Function	protein kinase activity	31	3.2	4.5E-3	3.7E-1

Molecular Function	GO Molecular Function	transcription coactivator activity	23	2.4	7.1E-3	4.9E-1
Molecular Function	GO Molecular Function	SUMO-specific endopeptidase activity	3	0.3	7.3E-3	4.9E-1
Molecular Function	GO Molecular Function	poly(A) RNA binding	76	7.6	7.7E-3	4.9E-1
Molecular Function	GO Molecular Function	ligase activity	24	2.5	9.6E-3	5.7E-1
Molecular Function	GO Molecular Function	helicase activity	11	1.1	1.0E-2	5.7E-1
Molecular Function	GO Molecular Function	ubiquinol-cytochrome-c reductase activity	4	0.4	1.2E-2	6.2E-1
Molecular Function	GO Molecular Function	core promoter binding	9	0.9	1.4E-2	7.2E-1
Molecular Function	GO Molecular Function	4 iron, 4 sulfur cluster binding	7	0.7	1.8E-2	8.4E-1
Molecular Function	GO Molecular Function	chaperone binding	10	1.0	2.0E-2	9.1E-1
Molecular Function	GO Molecular Function	protein C-terminus binding	17	1.8	2.1E-2	9.1E-1
Molecular Function	GO Molecular Function	polyubiquitin binding	5	0.5	2.6E-2	1.0E0
Molecular Function	GO Molecular Function	guanyl-nucleotide exchange factor activity	12	1.2	3.5E-2	1.0E0
Molecular Function	GO Molecular Function	damaged DNA binding	8	0.8	3.8E-2	1.0E0
Molecular Function	GO Molecular Function	ubiquitin binding	9	0.9	4.2E-2	1.0E0
Molecular Function	GO Molecular Function	nucleosome binding	4	0.4	4.3E-2	1.0E0
Molecular Function	GO Molecular Function	ubiquitin-protein transferase activity	25	2.6	4.4E-2	1.0E0
Molecular Function	GO Molecular Function	SUMO-specific protease activity	3	0.3	4.5E-2	1.0E0
Molecular Function	GO Molecular Function	ubiquitin protein ligase activity	16	0.7	5.0E-2	1.0E0
Molecular Function	GO Molecular Function	transferase activity	10	1.0	5.2E-2	1.0E0
Molecular Function	GO Molecular Function	magnesium ion binding	17	1.8	5.2E-2	1.0E0
Molecular Function	GO Molecular Function	endopeptidase activity	7	0.7	5.3E-2	1.0E0
Molecular Function	GO Molecular Function	ATP-dependent helicase activity	5	0.5	5.5E-2	1.0E0
Molecular Function	GO Molecular Function	phosphatidylethanolamine binding	3	0.3	5.8E-2	1.0E0
Molecular Function	GO Molecular Function	ubiquitin protein ligase activity involved in ERAD pathway	3	0.3	7.2E-2	1.0E0

Molecular Function	GO Molecular Function	peptide binding	7	0.7	7.5E-2	1.0E0
Molecular Function	GO Molecular Function	isomerase activity	4	0.4	8.6E-2	1.0E0
Molecular Function	GO Molecular Function	structural constituent of nuclear pore	4	0.4	8.6E-2	1.0E0
Molecular Function	GO Molecular Function	ubiquitin protein ligase binding	21	2.2	8.8E-2	1.0E0

Supplementary Table S4: Gene Ontology term enrichment (GOTERM)_ revealed several *FKBPs* correlated pathways and biological process

GO	Category	Term	Count	%	Log10(*P*)	Log10(q)
Biological Process	GO Biological Process	mitochondrial electron transport, NADH to ubiquinone	20	2.1	6.70E-13	1.90E-09
Biological Process	GO Biological Process	mitochondrial respiratory chain complex I assembly	20	2.1	1.10E-10	1.60E-07
Biological Process	GO Biological Process	mitochondrial translational elongation	21	2.2	4.60E-09	4.40E-06
Biological Process	GO Biological Process	mitochondrial translational termination	20	2.1	3.40E-08	2.40E-05
Biological Process	GO Biological Process	hydrogen ion transmembrane transport	13	1.4	4.00E-05	2.20E-02
Biological Process	GO Biological Process	mitochondrial electron transport, cytochrome c to oxygen	7	0.7	3.20E-04	1.50E-01
Biological Process	GO Biological Process	double-strand break repair	12	1.2	3.80E-04	1.50E-01
Biological Process	GO Biological Process	retrograde protein transport, ER to cytosol	6	0.6	4.20E-04	1.50E-01
Biological Process	GO Biological Process	mitochondrial translation	8	0.8	1.80E-03	5.00E-01
Biological Process	GO Biological Process	positive regulation of TOR signaling	7	0.7	1.80E-03	5.00E-01
Biological Process	GO Biological Process	translation	25	2.6	1.90E-03	5.00E-01
Biological Process	GO Biological Process	vascular endothelial growth factor receptor signaling pathway	11	1.1	2.90E-03	6.90E-01
Biological Process	GO Biological Process	intracellular protein transport	23	2.4	3.60E-03	7.70E-01
Biological Process	GO Biological Process	cellular response to DNA damage stimulus	21	2.2	3.80E-03	7.70E-01
Biological Process	GO Biological Process	protein phosphorylation	37	3.8	4.40E-03	8.30E-01
Biological Process	GO Biological Process	aerobic respiration	7	0.7	5.30E-03	8.90E-01
Biological Process	GO Biological Process	mitochondrial electron transport, ubiquinol to cytochrome c	5	0.5	5.40E-03	8.90E-01

Biological Process	GO Biological Process	mitochondrial respiratory chain complex III assembly	4	0.4	5.70E-03	9.00E-01
Biological Process	GO Biological Process	mitochondrial respiratory chain complex IV assembly	5	0.5	6.90E-03	9.10E-01
Biological Process	GO Biological Process	negative regulation of protein binding	9	0.9	7.00E-03	9.10E-01
Biological Process	GO Biological Process	DNA synthesis involved in DNA repair	7	0.7	7.10E-03	9.10E-01
Biological Process	GO Biological Process	DNA repair	22	2.3	7.10E-03	9.10E-01
Biological Process	GO Biological Process	regulation of signal transduction by p53 class mediator	14	1.5	8.90E-03	9.70E-01
Biological Process	GO Biological Process	transcription initiation from RNA polymerase II promoter	16	1.7	9.00E-03	9.70E-01
Biological Process	GO Biological Process	positive regulation of protein catabolic process	9	0.9	9.50E-03	9.70E-01
Biological Process	GO Biological Process	ER-associated ubiquitin-dependent protein catabolic process	9	0.9	9.50E-03	9.70E-01
Biological Process	GO Biological Process	protein ubiquitination involved in ubiquitin-dependent protein catabolic process	16	1.7	9.50E-03	9.70E-01
Biological Process	GO Biological Process	interstrand cross-link repair	8	0.8	1.00E-02	9.70E-01
Biological Process	GO Biological Process	transcription elongation from RNA polymerase II promoter	11	1.1	1.00E-02	9.70E-01
Biological Process	GO Biological Process	negative regulation of extrinsic apoptotic signaling pathway	7	0.7	1.10E-02	9.70E-01
Biological Process	GO Biological Process	cell cycle arrest	15	1.6	1.10E-02	9.70E-01
Biological Process	GO Biological Process	liver development	10	1	1.10E-02	9.70E-01
Biological Process	GO Biological Process	endoplasmic reticulum mannose trimming	4	0.4	1.10E-02	9.70E-01
Biological Process	GO Biological Process	negative regulation of apoptotic process	35	3.6	1.20E-02	1.00E+00
Biological Process	GO Biological Process	negative regulation of endoplasmic reticulum stress-induced intrinsic apoptotic signaling pathway	5	0.5	1.30E-02	1.00E+00
Biological Process	GO Biological Process	DNA damage checkpoint	6	0.6	1.50E-02	1.00E+00
Biological Process	GO Biological Process	intrinsic apoptotic signaling pathway	6	0.6	1.50E-02	1.00E+00
Biological Process	GO Biological Process	cell growth	8	0.8	2.10E-02	1.00E+00
Biological Process	GO Biological Process	protein ubiquitination	28	2.9	2.20E-02	1.00E+00
Biological Process	GO Biological Process	methylglyoxal catabolic process to D-lactate via S-lactoyl-glutathione	3	0.3	2.20E-02	1.00E+00
Biological	GO Biological	protein import into mitochondrial inner	3	0.3	2.20E-02	1.00E+00

Process	Process	membrane				
Biological Process	GO Biological Process	positive regulation of peptidyl-serine phosphorylation	9	0.9	2.30E-02	1.00E+00
Biological Process	GO Biological Process	endoplasmic reticulum unfolded protein response	7	0.7	2.30E-02	1.00E+00
Biological Process	GO Biological Process	cellular senescence	5	0.5	2.50E-02	1.00E+00
Biological Process	GO Biological Process	cell division	27	2.8	2.80E-02	1.00E+00
Biological Process	GO Biological Process	ER to Golgi vesicle-mediated transport	15	1.6	2.90E-02	1.00E+00
Biological Process	GO Biological Process	double-strand break repair via homologous recombination	9	0.9	3.10E-02	1.00E+00
Biological Process	GO Biological Process	protein sumoylation	12	1.2	3.20E-02	1.00E+00
Biological Process	GO Biological Process	DNA damage response, detection of DNA damage	6	0.6	3.20E-02	1.00E+00
Biological Process	GO Biological Process	regulation of Arp2/3 complex-mediated actin nucleation	3	0.3	3.30E-02	1.00E+00
Biological Process	GO Biological Process	pore complex assembly	3	0.3	3.30E-02	1.00E+00
Biological Process	GO Biological Process	cell aging	5	0.5	3.40E-02	1.00E+00
Biological Process	GO Biological Process	autophagy	13	1.4	3.40E-02	1.00E+00
Biological Process	GO Biological Process	macroautophagy	9	0.9	3.50E-02	1.00E+00
Biological Process	GO Biological Process	dolichol-linked oligosaccharide biosynthetic process	4	0.4	3.60E-02	1.00E+00
Biological Process	GO Biological Process	protein maturation	4	0.4	3.60E-02	1.00E+00
Biological Process	GO Biological Process	DNA double-strand break processing	4	0.4	3.60E-02	1.00E+00
Biological Process	GO Biological Process	double-strand break repair via nonhomologous end joining	8	0.8	3.70E-02	1.00E+00
Biological Process	GO Biological Process	strand displacement	5	0.5	3.80E-02	1.00E+00
Biological Process	GO Biological Process	negative regulation of transforming growth factor beta receptor signaling pathway	8	0.8	3.90E-02	1.00E+00
Biological Process	GO Biological Process	leukocyte migration	12	1.2	4.10E-02	1.00E+00
Biological Process	GO Biological Process	mitotic nuclear division	20	2.1	4.20E-02	1.00E+00
Biological Process	GO Biological Process	protein polyubiquitination	16	1.7	4.20E-02	1.00E+00
Biological Process	GO Biological Process	protein O-linked mannosylation	4	0.4	4.30E-02	1.00E+00
Biological	GO Biological	response to reactive oxygen species	6	0.6	4.30E-02	1.00E+00

Process	Process					
Biological Process	GO Biological Process	regulation of establishment of endothelial barrier	3	0.3	4.40E-02	1.00E+00
Biological Process	GO Biological Process	phospholipid scrambling	3	0.3	4.40E-02	1.00E+00
Biological Process	GO Biological Process	peptidyl-serine phosphorylation	12	1.2	4.80E-02	1.00E+00
Biological Process	GO Biological Process	positive regulation of catalytic activity	9	0.9	4.80E-02	1.00E+00
Biological Process	GO Biological Process	regulation of focal adhesion assembly	4	0.4	5.00E-02	1.00E+00
Biological Process	GO Biological Process	retrograde vesicle-mediated transport, Golgi to ER	9	0.9	5.10E-02	1.00E+00
Biological Process	GO Biological Process	androgen receptor signaling pathway	6	0.6	5.20E-02	1.00E+00
Biological Process	GO Biological Process	positive regulation of protein phosphorylation	12	1.2	5.20E-02	1.00E+00
Biological Process	GO Biological Process	regulation of RNA splicing	5	0.5	5.40E-02	1.00E+00
Biological Process	GO Biological Process	negative regulation of cell death	7	0.7	5.50E-02	1.00E+00
Biological Process	GO Biological Process	retrograde transport, endosome to Golgi	8	0.8	5.60E-02	1.00E+00
Biological Process	GO Biological Process	extrinsic apoptotic signaling pathway	6	0.6	5.60E-02	1.00E+00
Biological Process	GO Biological Process	positive regulation of hormone secretion	3	0.3	5.70E-02	1.00E+00
Biological Process	GO Biological Process	protein desumoylation	3	0.3	5.70E-02	1.00E+00
Biological Process	GO Biological Process	translational elongation	4	0.4	5.80E-02	1.00E+00
Biological Process	GO Biological Process	positive regulation of cell growth	9	0.9	5.80E-02	1.00E+00
Biological Process	GO Biological Process	protein transport	28	2.9	6.00E-02	1.00E+00
Biological Process	GO Biological Process	regulation of transcription, DNA-templated	89	9.3	6.10E-02	1.00E+00
Biological Process	GO Biological Process	response to activity	6	0.6	6.10E-02	1.00E+00
Biological Process	GO Biological Process	histone H3 acetylation	6	0.6	6.10E-02	1.00E+00
Biological Process	GO Biological Process	microtubule cytoskeleton organization	8	0.8	6.30E-02	1.00E+00
Biological Process	GO Biological Process	protein folding	15	1.6	6.60E-02	1.00E+00
Biological Process	GO Biological Process	cellular response to ionizing radiation	5	0.5	6.60E-02	1.00E+00
Biological Process	GO Biological Process	cellular response to nerve growth factor stimulus	5	0.5	6.60E-02	1.00E+00

Biological Process	GO Biological Process	mitotic nuclear envelope disassembly	6	0.6	6.70E-02	1.00E+00
Biological Process	GO Biological Process	DNA duplex unwinding	6	0.6	6.70E-02	1.00E+00
Biological Process	GO Biological Process	phosphatidylinositol biosynthetic process	7	0.7	6.80E-02	1.00E+00
Biological Process	GO Biological Process	DNA dealkylation involved in DNA repair	3	0.3	7.10E-02	1.00E+00
Biological Process	GO Biological Process	protein retention in ER lumen	3	0.3	7.10E-02	1.00E+00
Biological Process	GO Biological Process	nuclear mRNA surveillance	3	0.3	7.10E-02	1.00E+00
Biological Process	GO Biological Process	fat cell differentiation	8	0.8	7.10E-02	1.00E+00
Biological Process	GO Biological Process	RNA splicing	14	1.5	7.20E-02	1.00E+00
Biological Process	GO Biological Process	viral process	22	2.3	7.20E-02	1.00E+00
Biological Process	GO Biological Process	cellular response to reactive oxygen species	4	0.4	7.50E-02	1.00E+00
Biological Process	GO Biological Process	N-glycan processing	4	0.4	7.50E-02	1.00E+00
Biological Process	GO Biological Process	I-kappaB kinase/NF-kappaB signaling	7	0.7	7.70E-02	1.00E+00
Biological Process	GO Biological Process	protein stabilization	12	1.2	7.70E-02	1.00E+00
Biological Process	GO Biological Process	response to endoplasmic reticulum stress	8	0.8	8.00E-02	1.00E+00
Biological Process	GO Biological Process	negative regulation of protein phosphorylation	7	0.7	8.20E-02	1.00E+00
Biological Process	GO Biological Process	proteasome-mediated ubiquitin-dependent protein catabolic process	16	1.7	8.30E-02	1.00E+00
Biological Process	GO Biological Process	cellular response to amino acid stimulus	6	0.6	8.30E-02	1.00E+00
Biological Process	GO Biological Process	cellular component disassembly involved in execution phase of apoptosis	4	0.4	8.40E-02	1.00E+00
Biological Process	GO Biological Process	positive regulation of axonogenesis	4	0.4	8.40E-02	1.00E+00
Biological Process	GO Biological Process	regulation of keratinocyte differentiation	3	0.3	8.60E-02	1.00E+00
Biological Process	GO Biological Process	protein deglycosylation	3	0.3	8.60E-02	1.00E+00
Biological Process	GO Biological Process	bleb assembly	3	0.3	8.60E-02	1.00E+00
Biological Process	GO Biological Process	DNA replication	13	1.4	8.70E-02	1.00E+00
Biological Process	GO Biological Process	lung alveolus development	5	0.5	8.70E-02	1.00E+00
Biological	GO Biological	histone acetylation	5	0.5	8.70E-02	1.00E+00

Process	Process					
Biological Process	GO Biological Process	cellular response to insulin stimulus	8	0.8	8.90E-02	1.00E+00
Biological Process	GO Biological Process	mitochondrion organization	8	0.8	8.90E-02	1.00E+00
Biological Process	GO Biological Process	phospholipid translocation	4	0.4	9.40E-02	1.00E+00
Biological Process	GO Biological Process	membrane protein ectodomain proteolysis	4	0.4	9.40E-02	1.00E+00
Biological Process	GO Biological Process	positive regulation of protein complex assembly	4	0.4	9.40E-02	1.00E+00
Biological Process	GO Biological Process	regulation of cytokinesis	4	0.4	9.40E-02	1.00E+00
Biological Process	GO Biological Process	positive regulation of bone mineralization	5	0.5	9.50E-02	1.00E+00
Biological Process	GO Biological Process	negative regulation of protein K48-linked deubiquitination	2	0.2	9.70E-02	1.00E+00
Biological Process	GO Biological Process	dendritic spine organization	2	0.2	9.70E-02	1.00E+00
Biological Process	GO Biological Process	negative regulation of ubiquitin-specific protease activity	2	0.2	9.70E-02	1.00E+00
Biological Process	GO Biological Process	protein dephosphorylation	11	1.1	9.80E-02	1.00E+00
Biological Process	GO Biological Process	cellular response to oxidative stress	7	0.7	9.90E-02	1.00E+00

Supplementary Table S5: Gene Ontology term enrichment (GOTERM)_ revealed several *FKBPs* correlated pathways and cellular components

GO	Category	Term	Count	%	Log10(*P*)	Log10(q)
Cellular components	GO Cellular components	mitochondrial inner membrane	68	7.1	8.80E-17	5.10E-14
Cellular components	GO Cellular components	mitochondrial respiratory chain complex I	20	2.1	5.30E-13	1.60E-10
Cellular components	GO Cellular components	nucleoplasm	216	22.5	1.60E-12	3.20E-10
Cellular components	GO Cellular components	mitochondrion	113	11.7	1.10E-08	1.60E-06
Cellular components	GO Cellular components	membrane	158	16.4	6.60E-07	7.70E-05
Cellular components	GO Cellular components	mitochondrial large ribosomal subunit	12	1.2	1.60E-05	1.60E-03
Cellular components	GO Cellular components	integral component of endoplasmic reticulum membrane	16	1.7	1.70E-04	1.40E-02
Cellular components	GO Cellular components	ribosome	21	2.2	1.90E-04	1.40E-02
Cellular components	GO Cellular components	mitochondrial respiratory chain complex IV	6	0.6	2.60E-04	1.70E-02

Cellular components	GO Cellular components	nucleus	314	32.6	3.10E-04	1.70E-02
Cellular components	GO Cellular components	mitochondrial ribosome	8	0.8	3.20E-04	1.70E-02
Cellular components	GO Cellular components	nucleolus	65	6.8	5.60E-04	2.70E-02
Cellular components	GO Cellular components	endoplasmic reticulum	63	6.5	6.30E-04	2.80E-02
Cellular components	GO Cellular components	Golgi apparatus	65	6.8	6.60E-04	2.80E-02
Cellular components	GO Cellular components	mitochondrial intermembrane space	12	1.2	9.30E-04	3.60E-02
Cellular components	GO Cellular components	PML body	14	1.5	1.00E-03	3.70E-02
Cellular components	GO Cellular components	microtubule organizing center	18	1.9	1.40E-03	4.90E-02
Cellular components	GO Cellular components	Ragulator complex	4	0.4	2.10E-03	6.90E-02
Cellular components	GO Cellular components	nuclear inclusion body	5	0.5	2.90E-03	8.90E-02
Cellular components	GO Cellular components	intracellular membrane-bounded organelle	43	4.5	4.10E-03	1.20E-01
Cellular components	GO Cellular components	nuclear envelope	17	1.8	5.20E-03	1.40E-01
Cellular components	GO Cellular components	endoplasmic reticulum-Golgi intermediate compartment	10	1	5.90E-03	1.60E-01
Cellular components	GO Cellular components	focal adhesion	32	3.3	6.20E-03	1.60E-01
Cellular components	GO Cellular components	cytoplasm	290	30.1	8.80E-03	2.10E-01
Cellular components	GO Cellular components	melanosome	12	1.2	1.10E-02	2.50E-01
Cellular components	GO Cellular components	Sec61 translocon complex	3	0.3	1.40E-02	3.00E-01
Cellular components	GO Cellular components	respiratory chain	5	0.5	1.50E-02	3.20E-01
Cellular components	GO Cellular components	cytosol	189	19.6	1.60E-02	3.40E-01
Cellular components	GO Cellular components	site of double-strand break	6	0.6	1.70E-02	3.40E-01
Cellular components	GO Cellular components	mitochondrial matrix	26	2.7	2.00E-02	3.80E-01
Cellular components	GO Cellular components	endoplasmic reticulum membrane	57	5.9	2.00E-02	3.80E-01
Cellular components	GO Cellular components	nuclear speck	18	1.9	2.10E-02	3.80E-01
Cellular components	GO Cellular components	mitochondrial respiratory chain complex III	4	0.4	2.30E-02	4.00E-01
Cellular	GO Cellular	endoplasmic reticulum quality control	4	0.4	2.30E-02	4.00E-01

components	components	compartment				
Cellular components	GO Cellular components	chromosome, telomeric region	7	0.7	2.40E-02	4.10E-01
Cellular components	GO Cellular components	Golgi membrane	41	4.3	2.50E-02	4.10E-01
Cellular components	GO Cellular components	mitochondrial respiratory chain	4	0.4	2.90E-02	4.50E-01
Cellular components	GO Cellular components	transcription factor TFIID complex	6	0.6	3.00E-02	4.60E-01
Cellular components	GO Cellular components	lysosome	19	2	3.00E-02	4.60E-01
Cellular components	GO Cellular components	chromosome	11	1.1	3.40E-02	4.90E-01
Cellular components	GO Cellular components	intrinsic component of the cytoplasmic side of the plasma membrane	4	0.4	3.50E-02	4.90E-01
Cellular components	GO Cellular components	smooth endoplasmic reticulum	5	0.5	3.70E-02	5.00E-01
Cellular components	GO Cellular components	endoplasmic reticulum-Golgi intermediate compartment membrane	8	0.8	3.70E-02	5.00E-01
Cellular components	GO Cellular components	late endosome	12	1.2	3.80E-02	5.00E-01
Cellular components	GO Cellular components	ubiquitin ligase complex	11	1.1	4.00E-02	5.20E-01
Cellular components	GO Cellular components	contractile fiber	3	0.3	4.30E-02	5.20E-01
Cellular components	GO Cellular components	insulin-responsive compartment	3	0.3	4.30E-02	5.20E-01
Cellular components	GO Cellular components	bleb	3	0.3	4.30E-02	5.20E-01
Cellular components	GO Cellular components	perinuclear region of cytoplasm	41	4.3	4.90E-02	5.90E-01
Cellular components	GO Cellular components	extracellular exosome	157	16.3	5.10E-02	6.00E-01
Cellular components	GO Cellular components	cis-Golgi network	6	0.6	5.40E-02	6.20E-01
Cellular components	GO Cellular components	nuclear replication fork	3	0.3	5.60E-02	6.20E-01
Cellular components	GO Cellular components	mitochondrial inner membrane presequence translocase complex	3	0.3	6.90E-02	7.50E-01
Cellular components	GO Cellular components	flotillin complex	3	0.3	6.90E-02	7.50E-01
Cellular components	GO Cellular components	integral component of mitochondrial inner membrane	4	0.4	7.20E-02	7.60E-01
Cellular components	GO Cellular components	endosome membrane	15	1.6	7.30E-02	7.60E-01
Cellular components	GO Cellular components	ER membrane protein complex	3	0.3	8.40E-02	8.60E-01
Cellular components	GO Cellular components	cell body	7	0.7	8.90E-02	8.90E-01

Cellular components	GO Cellular components	vesicle membrane	4	0.4	9.10E-02	8.90E-01
Cellular components	GO Cellular components	mediator complex	5	0.5	9.10E-02	8.90E-01
Cellular components	GO Cellular components	ATF4-CREB1 transcription factor complex	2	0.2	9.60E-02	9.00E-01
Cellular components	GO Cellular components	Derlin-1 retrotranslocation complex	3	0.3	9.90E-02	9.00E-01
Cellular components	GO Cellular components	perinuclear endoplasmic reticulum	3	0.3	9.90E-02	9.00E-01

Supplementary Table S6: GeneGo Metacore showed that the co-expressed genes of FKBP family genes participated in several networks

#	Maps	p-value	Network Objects from Active Data
1	Ubiquinone metabolism	4.1E-19	NDUFB9, NDUFA8, NDUFB7, NDUFB3, COQ6, NDUFS8, NDUFA11, NDUFV1, NDUFS3, NDUFS7, NDUFS5, NDUFAB1, NDUFB1, NDUFA6, NDUFC2, NDUFC1, NDUFV3, NDUFA7, NDUFA2, NDUFB2, NDUFA3, NDUFA13, NDUFV2, NDUFA1, NDUFB4, NDUFA4, NDUFA10, NDUFB8, NDUFS6, NDUFB10
2	Translation_(L)-selenoaminoacids incorporation in proteins during translation	5.6E-05	Selenoprotein K, Selenoprotein H, GPX1, SelM, Selenoprotein W, GPX4, TXNRD2, Selenoprotein O, SEPX1 (Selenoprotein X1), SELS
3	Transcription_Negative regulation of HIF1A function	8.3E-05	Ubiquitin, Sirtuin3, Elongin B, PRDX2, Sirtuin7, OS-9, SART1, KLF2, ARD1, Sirtuin6, PRDX4, CHIP, SAT2
4	Oxidative stress_Role of Sirtuin1 and PGC1-alpha in activation of antioxidant defense system	8.4E-05	GSTP1, PRDX5, GPX1, SOD1, KEAP1, MT-TRX, AMPK beta subunit, TXNRD2, MSRA, Esrra, PPARGC1 (PGC1-alpha), UCP2
5	GTP-XTP metabolism	1.1E-04	ITPA, RRP41, NDPK C, CSL4, RPB5, NDPK D (mitochondrial), KGUA, RPB10, POLR2I, RPB6, POLR2G, RPA16, POLR2J, RRP43, POLR3K
6	ATP/ITP metabolism	1.3E-04	ITPA, RRP41, NDPK C, CSL4, RPB5, ACYP2, NDPK D (mitochondrial), RPB10, POLR2I, RPB6, POLR2G, ADSSL1, RPA16, POLR2J, RRP43, POLR3K, APRT, AK1
7	Epithelial cell apoptosis in COPD	2.0E-04	BAD, I-kB, PSMA1, PSMB6, KEAP1, DJ-1, Caspase-9, PUMA

**8**	Apoptosis and survival_IL-17-induced CIKS-dependent MAPK signaling pathways	2.6E-04	Ubiquitin, AP-1, IL-17RC, TRAF2, MEK3(MAP2K3), TAB1, MEK1/2, ABIN-2
**9**	CREB1-dependent transcription deregulation in Huntington's Disease	3.8E-04	COX VIIc, NDUFS3, GPX1, SOD1, COX VIa-1, PPARGC1 (PGC1-alpha), UCP2
**10**	Signal transduction_Adenosine A1 receptor signaling pathway	5.0E-04	BAD, I-kB, PKC, PLC-beta, H-Ras, MEK1/2, G-protein beta/gamma, CGRP, G-protein alpha-q/11, SFK, PLC-beta3
**11**	Naphthalene metabolism	5.1E-04	GSTP1, HYEP, AKR1C3, AKR1C4, GSTA1, AKR7A2, AKR1C1, CYP2E1, CYP2A6, GSTA2, ALDX, AKR1C2
**12**	NRF2 regulation of oxidative stress response	6.2E-04	GSTP1, Ubiquitin, Casein kinase II, beta chain (Phosvitin), PKC, SOD1, KEAP1, GSTA1, DJ-1, MafK, GSTA2
**13**	Apoptosis and survival_p53 and p73-dependent apoptosis	6.3E-04	BAD, Cystatin C, MM-1, ZNHIT1, OKL38, GPX1, Aif, DIP, Glyoxalase II, SIVA1, NOL3, Pin1, PUMA
**14**	CTP/UTP metabolism	7.3E-04	ITPA, RRP41, NDPK C, CSL4, RPB5, NDPK D (mitochondrial), RPB10, POLR2I, RPB6, POLR2G, RPA16, POLR2J, RRP43, POLR3K, AK1
**15**	Regulation of GSK3 beta in bipolar disorder	7.6E-04	FRAT1, Neutral sphingomyelinase, WNT, PP1-cat, H-Ras, DVL-1, LRP5, MEK1/2, Dsh
**16**	Histone deacetylases in Prostate Cancer	7.9E-04	Sirtuin4, Sirtuin3, HDAC11, Sirtuin7, HDAC8, HDAC10, Sirtuin6
**17**	Protein folding and maturation_Angiotensin system maturation	1.1E-03	Angiotensin I, Angiotensin (1-7), Angiotensin (1-9), Angiotensin III, Angiotensin (2-10), Angiotensinogen, Angiotensin IV, Angiotensin II, Cathepsin A
**18**	Glutathione metabolism	1.6E-03	GSTP1, 5-oxoprolinase, GPX1, GSTA1, GPX4, MGST3, MAAI, GSTA2, GGTL3, GSTA5, MGST2
**19**	Signal transduction_Relaxin family peptides signaling via RXFP3 and RXFP4 receptors	1.8E-03	p70 S6 kinases, AP-1, PKC, PLC-beta, MEK1/2, G-protein beta/gamma, G-protein alpha-q/11, SFK
**20**	N-Glycan biosynthesis p1	1.8E-03	DPM2 (reg), ALG10-A, ALG5, RIB1, OST48, RIB2, ALG1, DPM3 (reg), ALG12, GPT, ALG8
**21**	Chemotaxis_Lysophosphatidic acid signaling via GPCRs	1.9E-03	AP-1, PKC, PLC-beta, H-Ras, SIVA1, PKC-zeta, MEK1/2, G-protein beta/gamma, Rho GTPase, TAZ, Caspase-9, G-protein alpha-q/11, PLC-beta3, Cofilin, E3KARP (NHERF2), PREX1

**22**	Renin-Angiotensin-Aldosterone System	1.9E-03	MLK3(MAP3K11), Angiotensin I, PPAP2, PLC-beta, MEK3(MAP2K3), G-protein beta/gamma, Angiotensinogen, G-protein alpha-q/11, Angiotensin II, MDR1
**23**	HSP70 and HSP40-dependent folding in Huntington's disease	1.9E-03	Ubiquitin, PSMD1, HSP27, BAG-1, CHIP, SGTA
**24**	Protein folding_Membrane trafficking and signal transduction of G-alpha (i) heterotrimeric G-protein	3.0E-03	G-protein beta, PLC-beta, G-protein gamma, Rap1GAP1, G-protein beta/gamma
**25**	Apoptosis and survival_IL-17-induced CIKS-dependent NF-kB signaling and mRNA stabilization	3.6E-03	Ubiquitin, I-kB, IL-17RC, TRAF3, TRAF2, TAB1
**26**	Immune response_Innate immune response to RNA viral infection	3.6E-03	I-kB, TRAF3, IRF7, IRF3, TANK, TRADD
**27**	Regulation of degradation of wtCFTR	3.8E-03	Ubiquitin, RNF5, UFD1, HSPBP1, CHIP
**28**	Signal transduction_Adenosine A3 receptor signaling pathway	4.3E-03	BAD, PKC, PLC-beta, H-Ras, MEK1/2, G-protein beta/gamma, Rho GTPase, G-protein alpha-q/11
**29**	CHDI_DEGs from Replication data_Causal network	4.6E-03	I-kB, LTBR(TNFRSF3), PKC, TRAF2, WNT, PP1-cat, H-Ras, LRP5, CtBP, G-protein alpha-q, Dsh
**30**	Apoptosis and survival_Endoplasmic reticulum stress response	4.6E-03	NFYC, EDEM2, KEAP1, TRAF2, XBP1, ATF-4, PPARGC1 (PGC1-alpha), Caspase-9, TFEB
**31**	Development_BMP7 in brown adipocyte differentiation	4.8E-03	COX IV-1, TAB1, PPARGC1 (PGC1-alpha), CtBP, Angiotensinogen, CtBP1, COX Vb
**32**	Autocrine Somatotropin signaling in breast cancer	4.8E-03	PDF, GPX1, SOD1, TFF3, H-Ras, MEK3(MAP2K3), MEK1/2
**33**	Immune response_C5a signaling	5.5E-03	BAD, I-kB, AP-1, PKC, PLC-beta, MEK1/2, G-protein beta/gamma, PREX1
**34**	Apoptosis and survival_BAD phosphorylation	7.4E-03	BAD, G-protein alpha-s, H-Ras, PP1-cat alpha, MEK2(MAP2K2), G-protein beta/gamma, p70 S6 kinase2
**35**	Apoptosis and survival_Role of PKR in stress-induced apoptosis	7.9E-03	I-kB, TRAF3, TRAF2, TARBP2, ATF-4, NFKBIB, IRF3, MSK2
**36**	ENaC regulation in normal and CF airways	7.9E-03	G-protein alpha-s, PLC-beta, beta-ENaC, alpha-ENaC, Prostasin, G-protein beta/gamma, G-protein alpha-q/11, Furin
**37**	Immune response_Substance P-stimulated expression of	8.4E-03	I-kB, CCL13, PLC-beta, Substance P receptor, MEK1/2, G-protein beta/gamma, G-protein alpha-q

	proinflammatory cytokines via MAPKs		
**38**	Bipolar Disorder	8.4E-03	BAD, G-protein alpha-s, PKC, PLC-beta, XBP1, WNT, PKC-zeta, G-protein beta/gamma, G-protein alpha-q, IMPA2
**39**	CFTR folding and maturation (normal and CF)	8.8E-03	MA1B1, HSPBP1, BAG-1, ERp29, FKBP8
**40**	Immune response _CCR3 signaling in eosinophils	9.2E-03	Profilin I, Profilin, CCL13, p22-phox, H-Ras, MRLC, MEK2(MAP2K2), PKC-zeta, G-protein beta/gamma, Cofilin
**41**	Role of TLR signaling in skin sensitization	9.5E-03	I-kB, AP-1, TRAF3, HSP27, MEK3(MAP2K3), TAB1, IRF3
**42**	Role of mevalonate pathway in multiple myeloma	9.5E-03	MVD, H-Ras, MEK1/2, IDI2, PMVK, RhoB, MVK
**43**	Oxidative stress_ROS-mediated activation of MAPK via inhibition of phosphatases	9.7E-03	SOD1, PP2A structural, PP1-cat, MEK3(MAP2K3), MEK1/2, PR65-alpha
**44**	Development_PTHR1 in bone and cartilage development	1.0E-02	BAD, G-protein alpha-s, PKC, PLC-beta, Osteocalcin, PP1-cat, ATF-4, MEK1/2, G-protein alpha-q/11, SOX9
**45**	Putative pathways of activation of monoclonal protein secretion in multiple myeloma	1.0E-02	SSR-delta, XBP1, ARMET, SRP-alpha, Cyclophilin B
**46**	Transcription_Transcription regulation of aminoacid metabolism	1.0E-02	AP-1, PKC, KEAP1, MafK, HMBS
**47**	Role of neuropeptides in pathogenesis of SCLC	1.1E-02	PKC, PLC-beta, H-Ras, Substance P receptor, MEK1/2, G-protein beta/gamma, G-protein alpha-q, G-protein alpha-q/11, Neuromedin B
**48**	Signal transduction_Muscarinic acetylcholine receptors signaling to second messengers	1.1E-02	PKC, PPAP2, PLC-beta, H-Ras, FAAH, G-protein beta/gamma, G-protein alpha-q, G-protein alpha-q/11, PLA2, ARF1
**49**	Development_G-protein-mediated regulation of MAPK-ERK signaling	1.2E-02	G-protein alpha-s, PLC-beta, Rap1GAP1, H-Ras, MEK2(MAP2K2), G-protein beta/gamma, G-protein alpha-q/11
**50**	Role of GIP in pathogenesis of type 2 diabetes	1.2E-02	BAD, Ubiquitin, G-protein alpha-s, CaMKK, MEK3(MAP2K3), MEK1/2, Caspase-9
